# Monitoring of noble, signal and narrow-clawed crayfish using environmental DNA from freshwater samples

**DOI:** 10.1371/journal.pone.0179261

**Published:** 2017-06-27

**Authors:** Sune Agersnap, William Brenner Larsen, Steen Wilhelm Knudsen, David Strand, Philip Francis Thomsen, Martin Hesselsøe, Peter Bondgaard Mortensen, Trude Vrålstad, Peter Rask Møller

**Affiliations:** 1 Section for Evolutionary Genomics, Natural History Museum of Denmark, University of Copenhagen, Universitetsparken 15, Copenhagen Ø, Denmark; 2 Norwegian Veterinary Institute, Sentrum, Oslo, Norway; 3 Centre for GeoGenetics, Natural History Museum of Denmark, University of Copenhagen, Øster Voldgade 5–7, Copenhagen, Denmark; 4 Amphi Consult ApS, Niels Jernes Vej 10, Aalborg Øst, Denmark; 5 Eurofins Miljø A/S, Ladelundsvej 85, Vejen, Denmark; University of Hyogo, JAPAN

## Abstract

For several hundred years freshwater crayfish (Crustacea—Decapoda—Astacidea) have played an important ecological, cultural and culinary role in Scandinavia. However, many native populations of noble crayfish *Astacus astacus* have faced major declines during the last century, largely resulting from human assisted expansion of non-indigenous signal crayfish *Pacifastacus leniusculus* that carry and transmit the crayfish plague pathogen. In Denmark, also the non-indigenous narrow-clawed crayfish *Astacus leptodactylus* has expanded due to anthropogenic activities. Knowledge about crayfish distribution and early detection of non-indigenous and invasive species are crucial elements in successful conservation of indigenous crayfish. The use of environmental DNA (eDNA) extracted from water samples is a promising new tool for early and non-invasive detection of species in aquatic environments. In the present study, we have developed and tested quantitative PCR (qPCR) assays for species-specific detection and quantification of the three above mentioned crayfish species on the basis of mitochondrial cytochrome oxidase 1 (*mtDNA-CO1*), including separate assays for two clades of *A*. *leptodactylus*. The limit of detection (LOD) was experimentally established as 5 copies/PCR with two different approaches, and the limit of quantification (LOQ) were determined to 5 and 10 copies/PCR, respectively, depending on chosen approach. The assays detected crayfish in natural freshwater ecosystems with known populations of all three species, and show promising potentials for future monitoring of *A*. *astacus*, *P*. *leniusculus* and *A*. *leptodactylus*. However, the assays need further validation with data 1) comparing traditional and eDNA based estimates of abundance, and 2) representing a broader geographical range for the involved crayfish species.

## Introduction

Crayfish (Decapoda—Astacidea) are key species in freshwater ecosystems [1]. In Europe, all five indigenous crayfish species (ICS) are in decline and the number of non-indigenous crayfish species (NICS) (following abbr. by [2]) now exceeds the number of indigenous species [3, 2]. If we are to protect the native European species, an overview of both the ICS and NICS distributions is needed. Monitoring of freshwater crayfish is carried out in several countries in Europe [4]. In all the Nordic countries, the native noble crayfish *Astacus astacus* (Linnaeus, 1758) is heavily threatened by the invasive, signal crayfish *Pacifastacus leniusculus* (Dana, 1852) originally introduced from North America. In Denmark, *A*. *astacus* is also threatened by the narrow-clawed crayfish *Astacus leptodactylus* (Eschscholtz, 1823) which originates from south-eastern Europe and is known to displace other ICS when outside its natural distribution [5, 2]. Also in Finland, *A*. *leptodactylus* is introduced outside its natural distribution [3]. Monitoring programs targeting crayfish by means of catch per unit effort (CPUE) are carried out in some of the Nordic countries (Norway, Sweden and Finland) where *A*. *astacus* has an important status both culturally and culinary. *Aphanomyces astaci* (Schikora, 1906), a pathogenic oomycete causing crayfish plague which is lethal to *A*. *astacus* [6], has been spread all over Europe by invasive North American crayfish and specifically with *P*. *leniusculus* in the North. Thus, there is an urgent need for early detection of non-indigenous crayfish species and for monitoring of *A*. *astacus*. Traditional monitoring methods include traps [7], hand nets and more rarely snorkelling [8]. A new tool for managing aquatic organisms is the use of environmental DNA (eDNA) [9, 10, 11] in the water for detection and relative quantification of the species [12, 13]. Detection of species in aquatic habitats by means of eDNA depends on excretion or emission of cells containing DNA (e.g. in faeces, urine, mucus, gametes and epidermal cells) [14], and the use of either species-specific genetic markers for direct qPCR-based detection [13], or general markers for meta-barcoding [15, 9]. For aquatic animals, there has been a great focus on eDNA monitoring of vertebrates, primarily fish and amphibians [16, 15, 13], but there are also an accumulating number of studies exploring eDNA detection of invertebrates [17, 18, 13]. In microbiology, the principle of detecting and quantifying DNA by molecular means directly from the environment has been widespread for decades, and is today state of the art for any biodiversity surveys, pathogen surveillance and other monitoring purposes [19, 20, 21]. Tréguier et al. [22], Dougherty et al. [23] and Ikeda et al. [24] investigated the potential of eDNA to detect crayfish, and succeeds in detecting the invasive red swamp crayfish *Procambarus clarkii* (Girard, 1852) in several lakes in France, rusty crayfish *Orconectes rusticus* (Girard, 1852) in upper Midwest, USA, and the endangered Zarigani *Cambaroides japonicus* (De Haan, 1841), in streams in Japan, respectively. A recent study by Larson et al. [25] detects eDNA from both *O*. *rusticus* and *P*. *leniusculus* in large lakes in California and Nevada, USA. However the assay for *P*. *leniusculus* is not completely species specific. Other studies on crustaceans, however, found that the success rate in general is lower for decapods compared to fish and amphibians [26].

In the present study, we developed quantitative real-time PCR (qPCR) assays for species-specific detection of the three freshwater crayfish species found in nature in northern Europe, and investigated the potential of using eDNA detection as a monitoring tool. The assays were tested in two different laboratories in Denmark and Norway, using slightly different but comparable water sampling procedures and approaches for qPCR analysis. This enabled a rigorous test of the qPCR assays between laboratories and eDNA monitoring procedures. Our aim was to evaluate the effectiveness of our qPCR assays through analyses of water samples originating from natural crayfish locations from Denmark, Norway and Finland, and also to compare two different recommendations for making qPCR standards and determining detection and quantification limits for qPCR assays.

## Materials and methods

### Crayfish reference material

We used the identification keys by Füreder and Machino [27] and Souty-Grosset et al. [4] to identify crayfish specimens collected, by trapping during summer and fall 2014, on Zealand, Denmark (Table A in S1 File). These were subsequently preserved as museum material (Table A S1 File) museum abbreviation codes follow Fricke and Eschmeyer [28]. To minimize erroneous identification of crayfish, validated identification keys that abstain from using colour patterns should preferably be used [27] coupled with analysis of genetic markers. A cube of approximately 0.5 cm^2^ of tail muscles were collected from each specimen for sequence analysis, allowing us to key nucleotide sequence variation with morphological variation and match with the already known phylogenetic structures of European crayfish [29, 30]. For *A*. *leptodactylus* we followed the taxonomy by Akhan et al. [29].

The identity of the collected vouchered specimens was confirmed by sequencing the *mtDNA-CO1* barcode region. This was particular important for *A*. *leptodactylus*, which is a species complex of three subclades [29, 31]. Genomic DNA was extracted using the commercial Qiagen Blood & Tissue kit following the DNeasy quick-start protocol, followed by PCR using the broad range invertebrate primers HCO2198 and LCO1490 [32]. (PCR-setup details are listed in Text C in S1 File). Separated laboratory rooms were used for pre- and post-PCR procedures. Negative controls for each PCR setup included two DNA extraction blank controls and two PCR blank controls. Purification and sequencing of amplicons was performed commercially by Macrogen Europe (Amsterdam, The Netherlands, www.macrogen.com).

Sequences obtained from crayfish specimens were trimmed and visually inspected in Geneious v. R7.1.7, and consensus sequences (Accession numbers MF288079–MF288089; Table A in S1 File) from each individual specimen were aligned using the MAFFT algorithm [33] together with NCBI Genbank retrieved sequences of the same species. A neighbour joining analysis [34] was performed with 1000 bootstrap pseudoreplicates with *P*. *leniusculus* as outgroup (Figure A in S1 File). All samples were collected under permission by the Danish Environmental Agency (Permit: 2013-7330-000009) or in agreement with private owners (Lake Nydam).

### Study sites, eDNA sampling- and extraction procedures

#### Denmark

**Fig 1:**
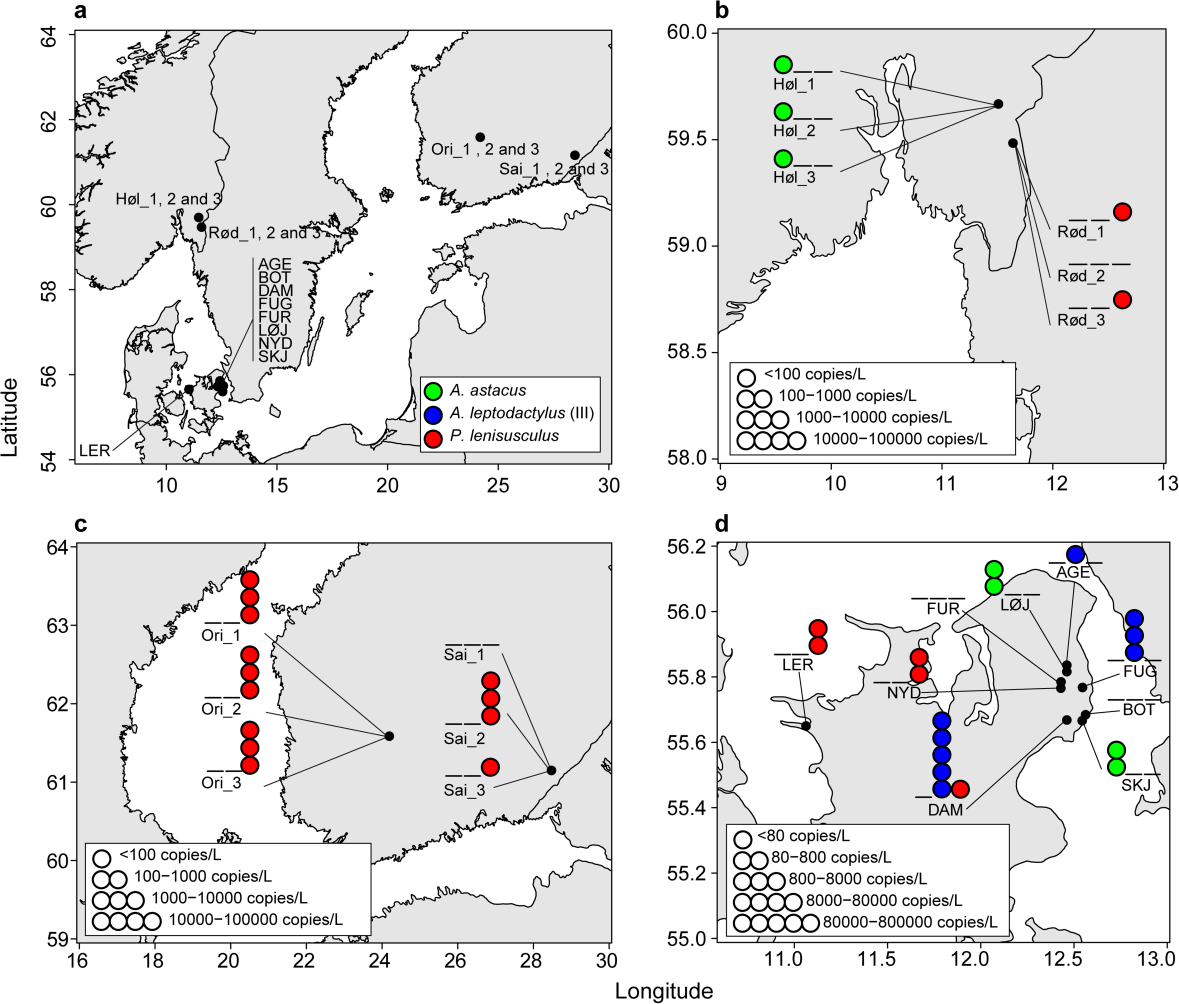
Map of the sampled locations, with detected species and levels of eDNA. A) Overview of sampling areas in Norway, Finland and Denmark. Abbreviated localities and coordinates are explained and given in Tables 1 and 2. Detection of *A*. *astacus*, *A*. *leptodactylus* clade III and *P*. *leniusculus* are marked with a blue, green and red circle, respectively. Detection of *A*. *leptodactylus* clade I have not been included in this figure. None detection of a species is marked with a line. Positive detections with 1 circle is given if the following criteria is fulfilled Ct-value < 41, technical replicate success > 2/4. Detection is quantified if the before mentioned criteria is fulfilled and eDNA concentration is > LOQ. The eDNA concentration levels are grouped into 5 groups with a 10 fold increase, and the LOD and LOQ values found in Figure C and Figure D in S1 File. B) Detection results from Norway. The eDNA concentration levels follows the LOD and LOQ found for the Norwegian and Finnish samples (Figure D in S1 File), with a tenfold increase between levels. C) Detection results from Finland. The eDNA concentration levels follows the LOD and LOQ found for the Finnish and Norwegian samples (Figure D in S1 File), with a tenfold increase between levels. D) Detection results from Denmark. The eDNA concentration levels follows the LOD and LOQ found for the Danish samples (Figure C in S1 File), with a tenfold increase between levels.

In Denmark, samples were taken from April to October 2015 in nine Danish waterbodies (Fig 1, Table 1 and Table A in S1 File). One filter sample was collected from the shore of each waterbody approximately 40 cm above the bottom substrate, with Lake Furesø as the exception. In Lake Furesø, water was collected and filtered during snorkelling on a stone reef. The sample volume varied from 0.5 L to 1.5 L (Table 1) between waterbodies, depending on water turbidity. Samples were filtered on-site using HSW Soft-Ject R 60 mL syringes and Sterivex^TM^-GP filter unit with a pore size of 0.22 μm, after filtration remaining water was removed. Filters were immediately stored in dark conditions and kept on ice during transport to the laboratory, where they were stored at -18°C. Extraction was done within 24 hours from sampling to minimize DNA degradation. Field and laboratory work was not carried out the same day to minimize the risk of contaminating samples. All extractions were performed in a separate room isolated from all PCR setups, and at least a day before any PCR setups were carried out. In line with recommendations provided by Deiner et al. [35] flow hood and cabinets were UV-light treated overnight prior to extractions. Surfaces and instruments were cleaned with a 5% bleach and 70% ethanol before and after laboratory work. Prior to extraction, the outside of the filter units were wiped with 5% bleach solution. Extraction was done using Qiagen DNeasy Blood & Tissue kit, following the supplied protocol with minor modifications [36]. Two DNA-extraction blank controls were included in each extraction setup.

**Table 1:** Danish eDNA collection localities from waters with known presence of *A*. *astacus*, *P*. *leniusculus* and *A*. *leptodactylus*.

Location/ filter sample	Locality, coordinates	Month	Species	Presence confirmed by	Population size, (95% C.L.)	Catch per unit effort (CPUE), individuals/hour)	Lake water filtered (mL) per individual filter	Positive qPCR replicates above Ct cut-off	Average level of eDNA, (copies/L filtered water (SD))
AGE	55°50'N; 12°28'E	Jun	*A*. *astacus*	Visual, trapping	N/A	0.34	1000	1/4	0
FUG	55°46'N; 12°33'E	Jun	*A*. *astacus*	No	N/A	N/A	500	1/4	0
LØJ	55°49'N; 12°28'E	Jun	*A*. *astacus*	Visual, trapping	N/A	0.23	750	4/4	203 (73)*
SKJ	55°40'N; 12°33'E	Jun	*A*. *astacus*	Visual	N/A	N/A	1000	4/4	103 (139)*
FUR	55°47'N; 12°26'E	Oct	*A*. *astacus*	Trapping	N/A	N/A	1000	0/4	-
DAM	55°40'N; 12°28'E	Apr	*A*. *astacus*	No			1000	1/4	0
BOT	55°41'N; 12°34'E	Jun	*A*. *leptodactylus* I	Visual	N/A	N/A	1500	4/4	N/A*
DAM	55°40'N; 12°28'E	Jun	*A*. *leptodactylus* I	Visual, trapping	N/A	N/A	1000	1/4	-
FUG	55°46'N; 12°33'E	Jun	*A*. *leptodactylus* I	Visual, trapping	N/A	N/A	500	0/4	-
NYD	55°46'N; 12°26'E	Apr	*A*. *leptodactylus* I	No	N/A	N/A	1000	1/4	N/A
FUR	55°47'N; 12°26'E	Oct	*A*. *leptodactylus* III	Visual, trapping	4334(2730–9770)^a^	N/A	1000	0/4	-
AGE	55°50'N; 12°28'E	Jun	*A*. *leptodactylus* III	Trapping	N/A	N/A	1000	2/4	902 (1100)*
DAM	55°40'N; 12°28'E	Jun	*A*. *leptodactylus* III	Visual, trapping	N/A	N/A	1000	4/4	87552 (47557)*
FUG	55°46'N; 12°33'E	Jun	*A*. *leptodactylus* III	Visual, trapping	N/A	N/A	500	4/4	1443 (4893)*
LER	55°39'N; 11°04'E	Jun	*A*. *leptodactylus* III	No	N/A	N/A	1000	0/4	-
NYD	55°46'N; 12°26'E	Apr	*A*. *leptodactylus* III	No	N/A	N/A	1000	1/4	0
DAM	55°40'N; 12°28'E	Apr	*P*. *leniusculus*	No	N/A	N/A	1000	2/4	<LOQ*
LER	55°39'N; 11°04'E	Jun	*P*. *leniusculus*	Trapping	N/A	N/A	1000	4/4	251 (150)*
NYD	55°46'N; 12°26'E	Oct	*P*. *leniusculus*	Visual, trapping	3827(2511–6506)	1.08–2.75	1000	4/4	126 (72)*

If known, crayfish population size is given in catch per unit effort (CPUE; average number of crayfish individuals per trap night). All filtered water samples were carried out three times per location. The average level of eDNA-target copies was inferred from the standard dilution series incorporated in each qPCR setup for each test for the presence of the three species of crayfish. All sampling was carried out in 2015. Collection localities are abbreviated: Lake Agersø (AGE), Lake in Copenhagen Botanical Garden (BOT), Lake Damhussøen (DAM), Lake Fuglsangssø (FUG), Lake Furesø (FUR), pond in Lerchenborg (LER), Lake Løjesø (LØJ), Lake Nydam (NYD), Lake Sankt Jørgenssø (SKJ). Detection below LOQ (5 copies per qPCR reaction for the Danish approach) is reported as <LOQ until the cut-off Ct-value of 41. A minimum of two out of four positive, are required to be interpreted as a positive detection by eDNA.

Results regarded as a positive detection are marked by a *.

^a^) Estimated pop. size on reef, June 2014.

#### Norway

Samples from Norway (Fig 1 and Table 2) were taken in the Halden watercourse. These were filtered on-site onto sterile glass fibre filters (AP25, 47 mm diameter; Millipore, Billerica, Massachusetts, USA) using a peristaltic pump, tygon tubing (Cole-Parmer, Illinois, USA) and a 47mm in line filter holder (Millipore). Samples were collected at two sites in the water course in July and September 2015: the Høland river (NOR–Høl) which is known to contain *A*. *astacus*, as is the upstream lakes; and the outlet of lake Rødnessjøen (NOR–Rød) where illegally introduced *P*. *leniusculus* were discovered with *A*. *astacus* in 2014 during the yearly monitoring of *A*. *astacus*. All handling of equipment was performed with clean disposable glows, and sterile filters were quickly added to the rinsed filter holder using an ethanol sterilized forceps. Within each site, water was pumped through the hose and filter holder for approximately 5 minutes prior to filtration at that specific site. Between different sites, the hose and filter holder was sterilized with 10% chlorine for a minimum of 15 minutes, then rinsed with a 10% sodium thiosulphate solution, prior to 5 minutes pumping of site-specific water before the filter was added. Ideally, 5 L water was filtered per filter sample (Table 2), but total filtered volume was sometimes lower due to high turbidity. Each filter was transferred with a clean forceps to a sterile falcon tube immediately after filtration, kept on ice during transport back to the laboratory, frozen for a minimum of 24 hours and freeze dried before eDNA extraction. DNA extraction followed Strand et al. [37] using a large volume CTAB extraction procedure for improved eDNA yield. Comparable laboratory precautionary measures as described for the Danish approach were followed. Blank controls included 1 extraction blank control per DNA extraction setup along with 1 laboratory environment control (tube with ddH2O left open during material processing and subsequent extraction). Both controls were included in subsequent qPCR tests.

**Table 2:** Norwegian and Finnish eDNA collection locations from waters with known presence of either *A*. *astacus* or *P*. *leniusculus*.

Location/ filter sample	Locality, coordinates	Month-year	Species	Presence confirmed by	Population estimate	Lake water filtered (L) per filter	Positive qPCR replicates above Ct cut-off	Estimated eDNA copies/L filtered water (SD)
FIN_Ori/1	61° 35'N 24°13'E	06–2010	*A*. *astacus*	Not present, *P*. *leniusculus* farm	0	5	0/4	0
FIN_Ori/2	“	“	*“*	“	“	5	0/4	0
FIN_Ori/3	“	“	*“*	“	“	2	1/4	0*
FIN_Ori/1	“	“	*P*. *leniusculus*	Farm owner	<1.3 ind/m^3^	5	2/4	*2391(282)*
FIN_Ori/2	“	“	*“*	“	“	5	4/4	*2322(344)*
FIN_Ori/3	“	“	*“*	“	“	2	4/4	*5003(1260)*
FIN_Sai/1	61° 09'N 28°30'E	06–2010	*A*. *astacus*	Not present, *P*. *leniusculus* lake	0	14	0/4	0**
FIN_Sai/2	“	“	“	“	“	14	0/4	0**
FIN_Sai/3	“	“	“	“	“	10	0/4	0**
FIN_Sai/1	“	“	*P*. *leniusculus*	Trapping	2.8 CPUE	14	1/4	0
FIN_Sai/2	“	“	“	“	“	14	4/4	*7561(543)*
FIN_Sai/3	“	“	“	“	“	10	2/4	<LOQ
NOR_Høl/1	59° 40'N 11°31'E	07–2015	*A*. *astacus*	Confirmed by local trappers	N/A	5	4/4	<LOQ
NOR_Høl/2	“	“	“	“	“	0.5	4/4	<LOQ
NOR_Høl/3	“	“	“	“	“	5	4/4	<LOQ
NOR_Høl/1	“	09–2015	“	“	“	1.5	1/4	0
NOR_Høl/2	“	“	“	“	“	0.5	2/4	<LOQ
NOR_Høl/3	“	“	“	“	“	1.5	3/4	<LOQ
NOR_Høl/1	“	07–2015	*P*. *leniusculus*	Assumed not present yet	0	5	0/4	0
NOR_Høl/2	“	“	“	“	“	0.5	0/4	0
NOR_Høl/3	“	“	“	“	“	5	0/4	0
NOR_Høl/1	“	09–2015	“	“	“	1.5	0/4	0
NOR_Høl/2	“	“	“	“	“	0.5	0/4	0
NOR_Høl/3	“	“	“	“	“	1.5	0/4	0
NOR_ Rød/1	59° 29'N 11°39'E	07–2015	*A*. *astacus*	Not present (eradicated)***	0	8	0/4	0
NOR_ Rød/2	“	“	“	“	“	4	0/4	0
NOR_ Rød/3	“	“	“	“	“	10	0/4	0
NOR_ Rød/1	“	09–2015	“	“	“	7	0/4	0
NOR_ Rød/2	“	“	“	“	“	8	0/4	0
NOR_ Rød/3	“	“	“	“	“	5	0/4	0
NOR_ Rød/1	“	07–2015	*P*. *leniusculus*	Confirmed by local trappers	0.16 CPUE	8	2/4	<LOQ
NOR_ Rød/2	“	“	“	“	“	4	0/4	0
NOR_ Rød/3	“	“	“	“	“	10	2/4	<LOQ
NOR_ Rød/1	“	09–2015	“	“	“	7	0/4	0
NOR_ Rød/2	“	“	“	“	“	8	0/4	0
NOR_ Rød/3	“	“	“	“	“	5	0/4	0

If known, crayfish population size is given in catch per unit effort (CPUE; average number of crayfish individuals per trap night) or in crayfish individuals per m^3^. Water were filtered at three different plots per location, thus results per filter sample is reported separately due to high eDNA copy number variability. Quantitative estimates of eDNA-target copies were inferred from the standard dilution series incorporated in each qPCR setup. The *Astast* and *Paclen* assays are tested for all samples regardless of species present. Detection below LOQ (10 copies per qPCR reaction for the Norwegian approach) is reported as <LOQ until the cut-off Ct-value of 41. DNA extracts from each filter is tested in a total of 4 qPCR replicates, 2 from concentrated DNA extract and 2 from 10x diluted extract. Quantitative estimates are only calculated from the *10x dilution qPCR replicates* due to observed inhibition in the replicates from concentrated DNA extracts. Collection localities are: *P*. *leniusculus* farm in Orivesi in Finland (FIN_Ori), Lake Saimaa, Finland (FIN_Sai), River Hølandselva of the Halden water course in Norway (NOR_Høl), Lake Rødenessjøen of the Halden water course in Norway (NOR_Rød).

* Detected in one sample at Ct 40.7 assumingly due to minor equipment contamination. Same equipment had been used in *A*. *astacus* localities (samples not analyzed here).

** Positive signal observed below Ct 41 in all samples assumingly due to minor equipment contamination, as explained above.

*** *A*. *astacus* population suffered from crayfish plague in winter to spring 2015 –assumed eradicated from the location

### Finland

The Finnish samples (Table 2) included for comparison represents eDNA extracts originating from Strand et al. [37]. The samples correspond to triplicates of an average of 12 L and 6.5 L (Table 2) water from Lake Saimaa (Fin–Sai) and a *P*. *leniusculus* farm in Orivesi (Fin–Ori), respectively. The filtration and DNA extraction procedures are described in Strand et al. [37], and follow largely the above description but with less careful decontamination procedures. Lake Saimaa had a known population of *P*. *leniusculus* with an estimated CPUE of 2.4 by the time of sampling [37]. Samples from the *P*. *leniusculus* farm in Orivesi were taken from an out-door pond (area 1000 m^2^, volume 1500 m^3^ [37]) stocked with *P*. *leniusculus* with an approximate population size of ~2000 individuals, yielding an estimate of 1.3 crayfish individuals/m^3^.

### Design of species-specific qPCR primers and probes

Crayfish *mtDNA-CO1* sequences from this study along with two *mtDNA-CO1* sequences obtained from NCBI Genbank (Table B in S1 File) for each of the 15 species of freshwater crayfish currently recognized in Europe [4, 3] were aligned using MAFFT, as described earlier. Species-specific primer and probe sequence motifs were identified in the mtDNA-region Tréguier et al. [22] used for species specific eDNA detection of *Procambarus clarkii* (Table 3). Visual comparison with *mtDNA-CO1* sequences from other crayfish species were used for selecting primer and probe sequence motifs with the least theoretical risk of cross-species amplification with non-target species (Table C and Figure B in S1 File).

**Table 3:** Four species-specific assays for *Astacus astacus*, *Pacifastacus leniusculus* and *Astacus leptodactylus* clade I and III. Assays are named after the first three letters in genus name and species name. *Astacus leptodactylus* assays are named after the clade numbers in Figure A in S1 File. reporter dye (Fam), black hole quenching dye (BHQ-1). Amplicon length of each assay was 65 base pairs.

Species	Primer/probe name	Primer/probe sequence	# bases
*Astacus astacus*	Astast_COI_F0336	GATTAGAGGAATAGTAGAGAG	21
*Astacus astacus*	Astast_COI_P0357	Fam-AGGAGTAGGGACAGGATGAACT-BHQ-1	22
*Astacus astacus*	Astast_COI_R0397	CTGATGCTAAAGGGGGATAA	20
*Pacifastacus leniusculus*	Paclen_COI_F0336	AACTAGAGGAATAGTTGAAAG	21
*Pacifastacus leniusculus*	Paclen_COI_P0357	Fam-AGGAGTGGGTACTGGATGAACT-BHQ-1	22
*Pacifastacus leniusculus*	Paclen_COI_R0397	CCGCTGCTAGAGGAGGATAA	20
*Astacus leptodactylus*, clade I	AstlepI_COI_F0336	AACTAGGGGTATAGTAGAGAG	21
*Astacus leptodactylus*, clade I	AstlepI_COI_P0357	Fam-AGGAGTAGGGACCGGATGAACT-BHQ-1	22
*Astacus leptodactylus*, clade I	AstlepI_COI_R0397	CTGATGCTAAAGGGGGATAA	20
*Astacus leptodactylus*, clade III	AstlepIII_COI_F0336	AACTAGAGGTATAGTAGAGGG	21
*Astacus leptodactylus*, clade III	AstlepIII_COI_P0357	Fam-GGGTGTAGGAACTGGATGAACC-BHQ-1	22
*Astacus leptodactylus*, clade III	AstlepIII_COI_R0397	CTGATGCTAGGGGAGGATAA	20

### Validation of the qPCR assay specificity

In the following, the four assays developed for *A*. *astacus*, *A*. *leptodactylus* clade I and III and *P*. *leniusculus* are referred to as “*Astast*”, “*AstlepI*”, “*AstlepIII*” and “*Paclen*”, respectively (Table 3). In addition to the theoretical specificity tests (*in silico*) described above, actual species specificity was tested (Text D in S1 File) for each assay by real time quantitative PCR (qPCR) screening of genomic DNA tissue extracts (Text B in S1 File) from the target species and sympatric non-target species. Negative controls (NTC) included two replicates of DNA extractions blank controls and two qPCR controls of ddH_2_O. The qPCR tests were performed on a Stratagene Mx3005P (Text D in S1 File). Primer and probe concentrations were optimized according to Text A in S1 File.

### qPCR approaches for crayfish eDNA detection and quantification

The optimized qPCR protocol used for crayfish eDNA detection and quantification for all assays is as follows: One qPCR reaction of 25 μL contained 10 μL TaqMan® Environmental Master Mix 2.0 (Life Technologies), 7 μL ddH_2_O, 1 μL of each primer and probe (1.2 μM and 0.1 μM, respectively) and 5 μL of extracted DNA template. Thermal settings includes 1) 5 min initial denaturation at 50°C, then 10 min at 95°C, 2) 50 cycles at 95°C for 30 s, annealing at 60°C for 1 min, 3) final extension for 10 min at 72°C.

We prepared standard dilution series of target DNA with known copy numbers, and established limit of detection (LOD) and quantification (LOQ) for the crayfish eDNA analyses. This was done using two different, but comparable, approaches in the two involved laboratories in Denmark (University of Copenhagen) and Norway (Norwegian Veterinary Institute) (Table E in S1 File). Below, these two approaches are described separately.

#### The Danish approach

The Danish approach followed the recommendations from Ellison et al. [38] for defining LOD and LOQ in eDNA studies. We used genomic DNA extracts already available from tissue samples of *A*. *astacus*, *P*. *leniusculus* and representatives of *A*. *leptodactylus* clade I and III (described above). For each of the four assays (*Astast*, *Paclen*, *AstlepI* and *AstlepIII*), the target-region was amplified with a conventional PCR set up using the same species-specific primers as for qPCR assays (Text E in S1 File). The resulting amplicons were checked on a 2% agarose gel before purification using the Qiagen QIAquick PCR purification kit following the supplied protocol. Concentrations of double stranded DNA (dsDNA) were measured on a Qubit 2.0 Fluorometer (Life Technologies) and the number of target copies was calculated from the specific molecular weight of each 65 bp sequence, using the Oligo-Calc engine [39]. From calculated concentrations of dsDNA, a stock concentration of 10^8^ copies/μL was prepared and stored at -20°C for subsequent qPCR tests–one stock for each species and clade. Less than 24 hours prior to each qPCR test on extractions from filtered water samples (see below), a dilution series of known concentrations ranging from 10^8^ copies/μL decreasing tenfold theoretically down to 0.1 copies/μL was prepared for each assay. In the following qPCR analysis performed on extractions from water samples, we included three replicates of each tenfold standard dilution. Using the recommendations put forward by Ellison et al. [38] LOD and LOQ was defined for each assay and LOD as the lowest concentration of the standard dilutions returning at least one positive replicate out of the three replicates prepared, and LOQ as the lowest concentration at which all three positive replicates were able to amplify on the purified target dsDNA [40, 41]. The standards and definitions were used in the further detection and quantification of crayfish eDNA in all Danish water samples. A standard curve was then prepared inferred from cycle threshold (Ct) and concentration (copies/qPCR reaction) for each assay, and plotted using R v3.2.4 [42], R-script available in S1 Appendix. This allowed us to estimate the number of target copies for three species of crayfish in different lakes. Optimized qPCR assays were tested on eDNA extracted from filters. In each assay, four replicate reactions were performed per sample. Standard dilutions with four replicates per tenfold dilution step ranging from 108-10-1 copies/μL dilutions, two positive DNA extraction controls, four negative PCR controls and two negative extraction controls were included in all qPCR runs.

From standard curves based on a dilution series of known concentrations, the concentration of eDNA in filtered water was calculated as:

$$C_{L}=\frac{C_{r}*(\frac{V_{e}}{V_{r}})}{V_{w}}$$

Where: C_L_ = copies of target-eDNA per volume lake water, C_r_ = copies of target eDNA per reaction volume, V_e_ = total elution volume after extraction, V_r_ = volume of eluted extract in used the qPCR reaction, V_w_ = volume of filtered lake water. Detection was considered reliable if minimum two of four qPCR replicates return positive amplification above LOD. qPCR replicates with no detection of eDNA were treated as zero (0) in the final calculation, if positive replicates originating from the same filtered sample were above LOQ, as recommended [38].

#### The Norwegian approach

The Norwegian approach followed recommendations for defining LOD and LOQ in qPCR assays used for diagnostic analyses of GMO and microbiological pathogens in food stuff, tissues and environmental samples e.g. [43, 19, 37]. Only the *Astast* and *Paclen* assays were tested in this approach. Genomic DNA was extracted from *A*. *astacus* and *P*. *leniusculus* tissue samples originating from the Norwegian lakes Steinsfjorden and Rødnessjøen, respectively, using QIAamp DNA Mini QIAcube kit (Text B in S1 File). A NanoDrop® ND-1000 Spectrophotometer (NanoDrop Technologies, Wilmington, DE) was used to measure the DNA concentration in ng/mL. Stock solutions of 50 ng/ μL genomic DNA from each species was and used to prepare a four-fold dilution series of 12 standard dilutions for each target species. In an initial qPCR test, 10 replicates of each standard dilution were run on a Stratagene Mx3005P. PCR-conditions followed the described protocol described, except that we here used the original procedure of 2 μL of extracted DNA and 10 μL ddH_2_O in the qPCR reaction. The qPCR data was analyzed in the MxPro software V.4.10 (Stratagene). For the standard showing signs of >100% detection rate, another 14 replicates were run (in total 24 replicates) to get a larger sample size for estimating DNA copy number in the standard dilutions on the basis of single molecule quantification (SIMQUANT) [43]. A template concentration of approximately 1 DNA copy (or PCR forming unit; PFU–used in Berdal et al [43] and Vrålstad et al [19]) per PCR volume will yield a positive:negative ratio of 7:3 (70% detection success)[43]. The copy number in the standard dilution closest to 70% detection rate were then calculated with most probable number (MPN) calculations according to Berdal et al [43]. The obtained copy number result was then used to calculate copy numbers in the other, more concentrated standards (Table D in S1 File). The LOD was established for each assay following the criteria that LOD is the lowest concentration that yields a probability of false negatives < 5% [43, 19], while LOQ was established using the same acceptance level as set for qPCR quantification of the crayfish plague pathogen *A*. *astaci* [19], with observed standard deviation (SD) < ±0.5 for the Ct-values.

Water samples originating from Norway and Finland were tested for *A*. *astacus* and *P*. *leniusculus* eDNA using the PCR-conditions for the *Astast* and *Paclen* assays as described above. The above mentioned standard dilutions were used to generate a standard curve for quantification of target DNA of *A*. *astacus* and *P*. *leniusculus* in the filtered water samples. These standards had estimated DNA copy numbers of 39321.6, 3932.16, 393.22, and 39.32 for both assays. Each DNA isolate from the filtered water samples was tested with 2x undiluted and 2x 10-fold diluted replicates, in total 4 replicates per sample. Following the principle that qPCR quantification is only possible in the absence of PCR inhibition and above the limit of quantification [44], the presence or absence of qPCR inhibition was controlled by calculating the difference in Ct values (ΔCt) between the undiluted and corresponding 10-fold diluted DNA replicates. In the absence of inhibition, the theoretical ΔCt value equals 3.32, but some variation is expected due to minor inaccuracies in amplification efficiency, manual pipetting, and other stochastic factors [45]. We accepted a variance level of 15%, allowing for quantification in samples where the ΔCt is 3.32 ± 0.5 (range = 2.82 to 3.82) between the undiluted and 10-fold diluted replicates. If ΔCt was within this range, DNA copy numbers were calculated as the mean of the undiluted replicates and the 10-fold diluted replicates, the latter multiplied by 10. In case of inhibition (if ΔCt <2.82) the estimated eDNA copy number was based on the 10-fold diluted DNA replicates alone, while if ΔCt > 3.82 (i.e. 10-fold dilution out of range), the estimation of eDNA copy number was based solely on the undiluted DNA replicates. From the eDNA copy number quantified in the qPCR reactions, the eDNA copy number per L water was estimated using the formula given above (C_L_=(C_r_* (V_e_/V_r_))/V_w_). If none or only one of the relevant replicates were detected below LOQ, further quantification was not performed and the result for the eDNA sample was reported as detected below LOQ (<LOQ). As a general role of caution, we suggest to introduce a cut-off was set at Ct 41 so that positive signals with a Ct value ≥ 41 are regarded unreliable and counted as not detected. However, we do report results also above Ct 41 to demonstrate when these issues arose in the current datasets. The standard curve inferred from cycle threshold (Ct) and concentration (copies/ qPCR reaction) for the *Astast* and *Paclen* assays were plotted in Sigmaplot 13.0.0.83 (Systat Software) together with results for the eDNA samples from Norway and Finland.

All plots and maps were prepared using R v.3.2.4 [42] and the packages: "fields" [46], "ggmap" [47], "mapdata" [48], "mapproj" [49], "maps" [50], "rworldmap" [51], "scales" [52], "sp" [53, 54], "RSvgDevice" [55], "TeachingDemos" [56]. The R-code used is available in the supplementary material (S1 Appendix).

## Results

### Real-time PCR assay optimization and specificity tests

A neighbour joining analysis of sequences obtained from the vouchered crayfish specimens and Genbank derived sequences (Table A in S1 File) confirmed that *A*. *leptodactylus* sequences from Denmark grouped in two of the three clades (I and III) (Figure A in S1 File) of the *A*. *leptodactylus* species complex inferred by Akhan et al. [29].

The developed species-specific qPCR assays for *A*. *astacus*, *A*. *leptodactylus* clade I and III, and *P*. *leniusculus* target the same part of the *mtDNA-CO1* region as Tréguier et al. [22] applied for *Procambarus clarkii* (Table 3). All four assays were tested using PCR (without probe) and qPCR, on 24 DNA extracted and vouchered tissue samples. The assays successfully amplified the focal species or subclades, with no cross amplification arising from non-target species. The number of mismatches between the sequences from other species and the species-specific primers and probes ranged between 6 and 15 base pairs (Table C, Figure B in S1 File). Optimization of primer and probe concentrations showed the same results for both *Astast* and *Paclen* assays. Both forward and reverse primers had the lowest Ct-value at a final reaction concentration of 1.2 μM. The probe performed best at a final reaction concentration of 0.1 μM. These results were extrapolated to both *AstlepI* and *AstlepIII* assays.

### Crayfish eDNA detection and quantification

#### The Danish approach

For the approach following Ellison et al. [38], we estimated LOD and LOQ for each assay and found it to be same for all three species, 5 copies/PCR reaction equal to 1 copy/μL extract (Figure C and Figure D in S1 File).

The species-specific qPCR assays successfully detected the expected crayfish species in water samples from seven out of nine investigated lakes in Denmark (Fig 1D). *A*. *astacus* was detected in two Danish lakes (Fig 1D and Table 1) where the species had been observed with traditional methods (i.e. trapping and visual observation). Trapping, snorkelling and eDNA-detection all failed to detect *A*. *astacus* in Lake Furesø (FUR) (Fig 1D and Table 1). However, in 2015 a local biologist caught a single specimen of *A*. *astacus* (ZMUC-CRU-8678) in an inlet to Lake Furesø. Representatives of *A*. *leptodactylus* clade I was detected but not quantified, in one Danish lake. The presence of the species has not yet been confirmed. *A*. *leptodactylus* clade III was detected in three lakes (Fig 1D and Table 1), and confirmed by traditional methods. Strangely, in Lake Furesø we were not able to detect *A*. *leptodactylus* by eDNA, despite it was caught during snorkelling and trapping. This might be due to the occurrence of an algae bloom in Lake Furesø during the time of sampling, which is known to inhibit eDNA detection [57]. The eDNA of *P*. *leniusculus* was detected in three lakes, where the species was confirmed with traditional methods in two of them. In Lake Damhussøen the presence of the *P*. *leniusculus* was not confirmed despite various efforts with snorkelling and trapping. The inlet of the Stream (Harrestrup å, has not yet been carefully examined for the presence of *P*. *leniusculus*.

Concentrations of eDNA above the LOQ were quantified (Fig 1D, Table 1 and Figure C in S1 File), and the highest average estimate obtained was 87500 eDNA copies/L filtered water from one location (Table 1). All PCR and extraction blank controls remained negative.

#### The Norwegian approach

For the approach following Berdal et al. [43] and Vrålstad et al [19], LOD was experimentally established as ~5 copies/PCR reaction. Here, the observed detection success for 22 replicates of a standard dilution corresponding to ~2.4 copies/PCR reaction was 90.9% for both the *Astast* and *Paclen* assays, and corresponded to Ct-values of 38.85 ±0.97 and 38.46 ±1.16, respectively (Table D in S1 File). The dynamic range of the *Astast* and *Paclen* assays were established between ~40 to 10 target DNA copies/PCR reaction (Figure D a-b in S1 File); thus LOQ was 10 copies/PCR reaction, where the assays still demonstrated acceptable repeatability with observed standard deviation (SD) < ±0.5 for the Ct-values (Figure D a-b in S1 File and Table D in S1 File).

The *Astast* and *Paclen* qPCR assays successfully detected the expected crayfish species also in water samples from Finland and Norway. PCR inhibition was commonly observed in qPCR reactions with undiluted DNA template. Thus, the quantitative DNA copy estimates were only based on qPCR replicates of the diluted (x10) DNA template, when detected above LOQ (10 copies/PCR reaction). Stored DNA extracts from water samples from 2010 collected in lake Saimaa and the *P*. *leniusculus* farm in Orivesi were positive for *P*. *leniusculus* eDNA, and yielded in some cases quantifiable data (Fig 1C and Table 2). In lake Saimaa with existing CPUE estimates of 2.4 [37], we detected ~7600 (±540) eDNA copies/L of *P*. *leniusculus mtDNA-CO1* in one of the three tested filter samples, while the remaining samples gave positive detection below LOQ. For the *P*. *leniusculus* farm with a known populations size of approximately 1.3 individuals/m^3^, we detected from ~2000–6000 eDNA copies/L in the filter samples (Table 2 and Figure D d in S1 File). Both these locations were only expected to host *P*. *leniusculus*. However, qPCR tests with *Astast*-assay yielded one positive qPCR replicate above the proposed cut-off value (Ct 40.7) for one filter sample from the *P*. *leniusculus* farm (Figure D d in S1 File). Further, weak signals after the proposed cut-off value (Ct 41) were observed for several of the filter samples from the *P*. *leniusculus* lake Saimaa (Table 2). We expect this is a result of minor carry-over contamination from the filtering equipment (houses and filter holders) that were used in both locations for *P*. *leniusculus* and *A*. *astacus* in Finland 2010, although disinfected between locations. Water samples collected in the river Hølandselva were positive for *A*. *astacus* and negative for *P*. *leniusculus* (Fig 1B, Table 2 and Figure Dc in S1 File). We have no CPUE data, but the location has been described as good *A*. *astacus* locality. Here, most samples are positive but below LOQ. In general, all sample replicates were positive in June, while less replicates yielded positive signals for the September samples at the same location (Table 2 and Figure Dc in S1 File). In Lake Rødnessjøen, a large population of *A*. *astacus* had gone extinct during the spring 2015 due to an outbreak of crayfish plague. Here, no trace of *A*. *astacus* was detected (even below Ct 41), while the illegally introduced *P*. *leniusculus* that had caused the outbreak was detected below LOQ in 2 filter samples from July. No positive signals were obtained for the corresponding samples in September (Fig 1B, Figure D c in S1 File and Table 2). A total of 960 trap nights returned 110 *P*. *leniusculus* and zero *A*. *astacus*, giving a CPUE of 0.12 *P*. *leniusculus* per trap night in lake Rødnessjøen (Øystein Toverud, personal communication).

## Discussion

### Performance of the crayfish eDNA detection systems

According to Parkyn [58] crayfish research needs to develop new and standardized methods to monitor and quantify the abundance of crayfish. The results of this study provide a basis for detecting crayfish by eDNA, and thereby enhance the opportunity for future improvement of crayfish monitoring and management.

We present four species specific qPCR assays that successfully detect eDNA of the focal crayfish species, *A*. *astacus*, *A*. *leptodactylus clade I & III and P*. *leniusculus*, in natural aquatic environments in Denmark, Norway and Finland. The assays target the same *mtDNA-CO1* region that Tréguier et al. [22] used for eDNA detection of the red swamp crayfish (*Procambarus clarkii*). A recently published qPCR assay for detection of *P*. *leniusculus* eDNA targets another region of *mtDNA-CO1* [25], but is less species-specific than our assay. It relies on confirmative Sanger sequencing after qPCR detection, which will not only increase analytical costs, but is also difficult to implement in cases of low eDNA copy numbers and/or mixed PCR templates.

We were able to detect crayfish in the sampled lakes with crayfish present, indicating that the assays are effective in detecting the presence or substantiate the absence of the species, but of course with uncertainties when the population size is very low. For example, in the Norwegian Lake Rødenessjøen, *P*. *leniusculus* had recently had been introduced in low densities (0.16 CPUE) and led to the eradication of a well-established *A*. *astacus* population. Here, the *Paclen* assay detected *P*. *leniusculus* eDNA below LOQ in two of six tested filter samples in total representing 42 L of filtered water, while no traces of *A*. *astacus* eDNA was found in the system 3–5 months after the eradication event. The high success rate with regards to detection in lakes with known presence of the species (100%) is considerably higher than Tréguier et al. [22] who found crayfish in 59% of 158 ponds, which is likely to be explained by the larger volume of water filtered (3*0.5–1.5 L versus 6*15mL), or the fact that more study sites were included in the study of Tréguier et al. [22]. Our results are more in line with Dougherty et al. [23] and Ikeda et al. [24], who got 100% detection with 10*0.25 L and 1*1 L, respectively.

Both for the Danish and Norwegian approaches, dilution series made either from purified dsDNA or genomic tissue derived mtDNA performed well for the establishment of standard curves for quantification of eDNA from water samples. Thus, we recommend that future attempts to quantify eDNA should be based on dilution series as described in this study. For the Danish approach following the recommendations from aquatic eDNA studies [40, 59], the estimated LOD and LOQ for all assays was 1 copy/μL extract which equals to 5 copies/qPCR reaction. In our study the LOD was equivalent to ~80 copies/L filtered water which was close to the LOD of approximately 62 copies/L seawater reported by Thomsen et al. [13] for the fish species *Platichthys flesus* (Linnaeus, 1758) and *Gasterosteus aculeatus* (Linnaeus, 1758). With the Norwegian approach following recommendations from food- and disease diagnostics (e.g. [60, 19, 61, 43]), we estimated the same LOD at 5 copies/PCR reaction, while the LOQ was experimentally established at 10 copies/PCR reaction. Since LOD and LOQ in the water samples depend on several variables, in particular filtered water volume, the LOD and LOQ per liter filtered water vary. In cases where 5L water is filtered, the LOD and LOQ was 100 and 200 copies/L water, respectively for the Norwegian approach.

### Defining LOD and LOQ

There are to our knowledge no standardized protocols for defining LOD and LOQ in eDNA studies, even though successful quantification of eDNA is the key to increased information about populations targeted by eDNA sampling. Several studies [62, 22, 41], used pure genomic DNA to estimate LOD and LOQ in ng/μL, thus, quantifying the amount of genomic DNA and not the targeted gene region. Furthermore, some studies e.g. [14] quantified eDNA in copies/μL, but provide no explanation on how these values were estimated. Among the better approaches presented so far for estimating LOD and LOQ in eDNA studies is from a dilution made from purified PCR product, as described both by Wilcox et al. [40] and Thomsen et al. [59], adopted in this study for the Danish samples. This lack of one standardized protocol to estimate LOD and LOQ, makes it difficult to compare both detection and quantification of eDNA between studies. Many issues remain to be addressed [9] before reliable quantification of biomass/individuals by eDNA using qPCR can be achieved; including Ct-cutoff values and percentage of positive replicates for defining true positives from background, as well as proper treatment of negative qPCR replicates [63]. In contrast to the eDNA studies, there are several strict statistically based standards for calculating LOD and LOQ for analytical qPCR used in food and disease diagnostics [60, 19, 61], with defined rules for acceptance levels of variance for quantification. In this study, we made an attempt to follow, at least in part, these stricter rules in the Norwegian approach, although we are less stringent when it comes to acceptance of variance in the natural water samples compared to what is accepted for quantification of DNA copy number of e.g. GMO in food samples [61]. We also suggest a cut-off at Ct 41, which is justified both by the increased probability of detecting false positives at higher Ct values [45] as well as a precautionary measure to exclude positive signals from minor carry over contamination. We detected minor traces of *A*. *astacus* eDNA at high Ct values (from ~41–46) in two *P*. *leniusculus* locations in Finland where the presence of *A*. *astacus* is excluded. This was most likely a result of carry over contamination when using the same filter holder and houses between *P*. *leniusculus* and *A*. *astacus* locations, despite that disinfection of the equipment between localities was performed. No detections beyond Ct 41 were observed in the Danish samples. This can indicate that disposable Sterivex filters, as used in for the Danish samples, have advantages with regards to field contamination risk, and/or that more thorough disinfection procedures or use of separate filter holders and houses between *A*. *astacus* and *P*. *leniusculus* localities minimize this risk.

### Challenges of eDNA monitoring

Several factors can obscure the relationship between eDNA and actual presence/absence (and especially abundance) of target organisms [63]. Among these are biases introduced with humic acids or humic substances that are coextracted with DNA in environmental samples, and that strongly inhibit enzymes used in the qPCR reactions and thus leading to underestimation of DNA quantities or false negatives [64]. Inhibition occurs regularly in eDNA studies [65, 16] Tests done for the Norwegian and Finnish waters samples in our study showed inhibition for the qPCR result for undiluted DNA template. Thus, a dilution of the template was necessary both for obtaining reliable positives and quantitative results. Other factors complicating eDNA monitoring are various degree of eDNA dilution and dispersion in different aquatic habitats [17, 66, 67], temporal and spatial variability in eDNA degradation due to factors such as microbial activity, water chemistry, temperature, UV, etc. [68, 69], as well as species-, season- and age-specific eDNA shedding rates. Furthermore, relation between the actual eDNA on-site and final eDNA signal recovered after qPCR analyses will be influenced by e.g. the amount of water sampled, the area covered, the amount of eDNA captured on the filter, the proportion of this eDNA subsequently obtained from DNA extraction, primer/probe affinity, and stochasticity in PCR etc.

A specific shortcoming of qPCR in eDNA studies is the fact that target species has to be known in advance for primer/probe systems to be designed. This limits the approach to areas with well-known diversity for which reference DNA sequences are available. Environmental DNA metabarcoding (high-throughput sequencing of PCR amplicons from generic primers), Taberlet et al. [10] has recently proven very successful for analysing species compositions and populations [13, 70, 15, 71, 72]. Nevertheless, generic primers can suffer from template competition and biased amplification as well as incomplete resolution of taxa to species level [73, 72]. In this regard, targeted qPCR has high specificity and sensitivity and is appropriate when the purpose of the study is a few target species of interest.

### Environmental DNA for abundance estimates

Several studies have explored whether a relationship between abundance and eDNA could be inferred [13, 14, 25, 57, 72, 74]. The amount of eDNA and the chances of positive eDNA detection depends on several variables [75], e.g. volume of water filtered, density and activity of the study organism, DNA excretion rate and inhibitors such as humic compounds, as mentioned above [66, 23, 68]. Tréguier et al [22] speculates that the exoskeleton of crayfish might inhibit the excretion of eDNA and thereby detection. Larson and colleagues [25] detected eDNA of *O*. *rusticus* and *P*. *leniusculus* in large lakes, but found a very poor agreement between eDNA copy number and estimates of crayfish relative abundance by baited trapping. Thus, previous studies [24, 25, 22, 23] and the results presented here, document that the eDNA methods are sensitive enough for detecting crayfish, but that meaningful quantification is difficult. In our study, we observe not only that PCR inhibition prevents meaningful quantification in some samples unless the DNA extracts are diluted, but also that heterogeneously distributed eDNA sources in the water prevent reliable quantitative estimates. Rather large standard deviation values are observed, and some estimates also vary from high (e.g. 7600 ±540) to below LOQ for samples taken within the same areas of the *P*. *leniusculus* in lake Saimaa. Clearly, substantial development and validation work remains before it is possible to evaluate if quantitative estimates of crayfish eDNA copy numbers will make sense compared to crayfish population estimates. However, we do observe a clear relationship between high eDNA quantities in the densely populated Finnish *P*. *leniusculus* farm (1.3 crayfish/m^3^) compared to the low detection success in Lake Rødenessjoen with very low densities of *P*. *leniusculus* (0.3 CPUE).

Another concern when trying to quantify crayfish is their benthic engineering behaviour, that might release eDNA from sediment to the water column. A recent study by Turner et al. [76] on bigheaded Asian carp *Hypophthalmichthys* spp, found that the top 2 cm of the sediment contained 8 to 1800 times higher concentrations of carp eDNA than water samples, probably due to settling of eDNA from the water column. Further, Turner et al. [76] detected carp eDNA in sediment up to 132 days after carp removal. It is therefore important that samples are taken close to the bottom, without suspending any substrate. In our study, the Norwegian and Finnish samples were collected 5–10 cm above the sediments, and water were always first pumped through the system in the absence of a filter to get rid of disturbed sediment residues in the water. Here we observe that eDNA from *A*. *astacus* is gone from the system only some months after an on-going crayfish plague outbreak that lead to their eradication. This strongly indicate that we did not detect preserved eDNA from sediments with this sample approach, and also confirm previous observations that the decay of eDNA in freshwater beyond the threshold of detectability happens quickly, making positive eDNA detection a likely sign of the contemporary presence of species and populations while signals from past populations are not detected.

## Conclusion

Here we successfully designed and tested four species-specific qPCR assays for detection of *A*. *astacus*, *P*. *leniusculus* and *A*. *leptodactylus*, both experimentally and in natural freshwater environments in Denmark, Norway and Finland. Our assays were able to detect eDNA from the known present species in ten lakes and one farm. Furthermore, we observed a trend that densely populated waters contained much higher crayfish eDNA copy numbers than waters with very low population estimates. However, our data material was too limited to statistically compare the quantified amount of eDNA with the crayfish population estimates obtained with traditional methods. Intriguingly, we also discovered that two genetic distinguishable clades of narrow-clawed crayfish are present in Denmark. Our results show promising potential for future monitoring and management of freshwater crayfish by means of eDNA.

## Supporting information

S1 File **Contains all Supporting Figures (A-B), Tables (A-F) and Text (A-E)**:
**Figure A. Neighbour joining tree showing relationship between sequences of *mtDNA-CO1* from *A*. *leptodactylus*, *A*. *astacus* and *P*. *leniusculus*.** For each branch, species and accession numbers/museum catalog numbers are shown. Three clades have been collapsed: the hexagon is accession numbers JQ421496-JQ421509 within clade I, the square represents clade II and the triangle represents accession numbers KC311416, KC789374-KC789393 within clade I. The star and circle represent *P*. *leniusculus* subspecies *klamathensis* and *trowbridgii*, respectively. *P*. *leniusculus* was used as outgroup and from 1000 bootstrap pseudo replicates, relevant bootstrap values above 60% are shown. All ZMUC-CRU specimens were collected for the present study, and are listed in Table A in S1 File with Genbank accession numbers.
**Figure B. An alignment of the *mt*DNA-CO1 65 base pair fragment, used in this assay, for each of the *Astacoidea* and *Parastacoidea* present in Europe.** Species and NCBI acc. number aligned using MAFFT algorithm in Geneious (*Astacus astacus* JN254670, JN254671; *Astacus leptodactylus* Clade I JQ421471, JQ421471; *A*. *leptodactylus* Clade II JQ421478, JQ421479; *A*. *leptodactylus* Clade III JQ421489, JQ421490; *Austropotamobius torrentium* AY667128, AM180946; *A*. *italicus* HM622614, AY121127; *A*. *pallipes* AY667114, AY667115; *Cherax destructor* KM039112, KJ950555; *Orconectes immunis* JF438005, JF438006; *O*. *limosus* JF437992, JF437993; *O*. *virilis* FJ608577, EU442743; *O*. *rusticus* AY701248, AY701249; *Pacifastacus leniusculus* JF437995, JF437995; *Procambarus clarkii* JN000900, JN000901; *Procambarus sp*. HM358011, KF033123). The species-specific primers and probes used in this study are mapped to the sequences where they have the best match. Forward primers (F0336) Reverse primers (R0397) are green and probes (P0357) are red. Agreements to consensus are marked with dots and disagreements to consensus are highlighted. The specific number of mismatches between primer-probes and sequences are described in Table C in S1 File.
**Figure C. Standard curve for dilution series and filtered water samples comparing the concentration of eDNA target (copies/qPCR reaction) with cycle threshold (Ct). The approach used by University of Copenhagen.** A) eDNA results along with standard curves for each of the three assays *A*. *astacus (Astacus astacus—Astast*), *A*. *leptodactylus* (*Astacus leptodactylus* clade III—*AstlepIII*) and *P*. *leniusculus* (*Pacifastacus leniusculus—Paclen*). Each standard curve for each of the three species is based on three replicates, the dotted vertical line represents both limit of detection (LOD) and limit of quantification (LOQ). Cut-off at Ct 41 is indicated for each plot. Abbreviated localities are explained in Table 2 and Tabel B in S1 File) eDNA results along with standard curve for *P*. *leniusculus*, with an efficiency of 95.8% and R^2^ of 0.998 (Y = -3.427*LOG(X) + 39.34). C) eDNA results along with standard curve for *A*. *astacus* with an efficiency of 97.4% and R^2^ of 0.994 (Y = -3.386*LOG(X) + 40.15). D) eDNA results along with standard curve for *A*. *leptodactylus*, with an efficiency of 88.78% and R^2^ of 0.987 (Y = -3.622*LOG(X) + 41.92).
**Figure D. Standard curves for dilution series and filtered water samples comparing the concentration of eDNA target (copies/qPCR reaction) with cycle threshold (Ct). The approach used by Norwegian Veterinary Institute.** A) Standard curve for *A*. *astacus* (Astast—*A*. *astacus*) with an efficiency of 97.3% and R^2^ of 0.995 (Y = -3.390*LOG(X) + 40.26). B) Standard curve for *P*. *leniusculus* (Paclen—*P*. *leniusculus*) with an efficiency of 93.98% and R^2^ of 0.996 (Y = -3.475*LOG(X) + 39.87). Both standard curves were based on 4-fold dilution series and 10 qPCR replicates per standard dilution 4x10^-3^–4x10^-9^, and 22 and 24 replicates for the standards 4x10^-10^ and 4x10^-11^, respectively. C) eDNA results along with standard for each qPCR run from filtered water samples for Astact (*A*. *astacus*) with an efficiency of 99.77% and R^2^ of 0.986 (Y = -3.328*LOG(X) + 40.67). D) eDNA results along with standard for each qPCR run from filtered water samples for Paclen (*P*. *leniusculus*) with an efficiency of 98.48 and R^2^ of 0.980 (Y = -3.359*LOG(x) + 39.85). Abbreviated localities are explained in Table A in S1 File. LOD (5 copies/PCR, LOQ (10 copies/PCR) and cut-off at Ct 41 are indicated.
**Table A: Danish collected vouchered museum specimens.** Sampling sites, museums ID number (Natural History Museum of Denmark) and corresponding NCBI GenBank accession numbers for some specimens.
**Table B: Nationality and NCBI Genbank accession numbers for mt-DNA CO1 sequences used in Neighbour Joining analysis presented in Figure A in S1 File.** Literature sited in this table:
Akhan S, Bektas Y, Berber S, Kalayci G. Population structure and genetic analysis of narrow-clawed crayfish *Astacus leptodactylus* populations in Turkey. Genetica. 2014;142:381–395. 10.1007/s10709-014-9782-5. Available from: http://dx.doi.org/10.1007/s10709-014-9782-5
Filipová L, Grandjean F, Chucholl C, Soes DM, Petrusek A. Identification of exotic North American crayfish in Europe by DNA barcoding. Knowledge and Management of Aquatic Ecosystems. 2011;(401):article 11. 10.1051/kmae/2011025. Available from: http://dx.doi.org/10.1051/kmae/2011025.
Jadan M, Coz-Rakovac R, Topic Popovic N, Strunjak-Perovic I. Molecular characterization of the Croatian noble crayfish (*Astacus astacus* L.) population based on sequences from mitochondrial 16S rRNA and COI genes; 2010. Unpublished.
Keskin E, Atar HH. DNA barcoding commercially important aquatic invertebrates of Turkey. Mitochondrial DNA. 2013;24:440–450. 10.3109/19401736.2012.762576. Available from: http://dx.doi.org/10.3109/19401736.2012.762576.
Maguire I, Podnar M, Jelic M, Štambuk A, Schrimpf A, Schulz H, et al. Two distinct evolutionary lineages of the *Astacus leptodactylus* species-complex (Decapoda: Astacidae) inferred by phylogenetic analyses. Invertebrate Systematics. 2014;28:117. 10.1071/is13030. Available from: http://dx.doi.org/10.1071/IS13030.
Schrimpf A, Schulz HK, Theissinger K, Pârvulescu L, Schulz R. The first large-scale genetic analysis of the vulnerable noble crayfish Astacus astacus reveals low haplotype diversity in central European populations. Knowledge and Management of Aquatic Ecosystems. 2011;(401):35. Available from: http://dx.doi.org/10.1051/kmae/2011065.
Soroka M, Swierczynski M, Cybulski C, Lubinski J. Phylogenitic relationships among the Polish genera of freshwater crayfishes (Decapoda); 2002. Unpublished.
**Table C. Species-specific primer-probe assays for *Astacus astacus*, *Pacifastacus leniusculus* and *Astacus leptodactylus* with number of mismatch in the alignment with various other species of *Astacoidea* and *Parastacoidea*.** Species abbreviations for qPCR assays are: *Astacus astacus* (*Astast*), *Pacifastacus leniusculus* (*Paclen*), *Astacus leptodactylus*, clade I (*AstlepI*) and *Astacus leptodactylus*, clade III (*AstlepIII*). Probes (i.e. Astast_CO1_P0357, Paclen_CO1_P0357, AstlepI_CO1_P0357 and AstlepIII_CO1_P0357) were modified with a FAM-dye at the 5’-end and a BHQ-1 at the 3’-end.
**Table D. Standard dilutions from genomic crayfish DNA.** Standard curves were established from several calibration points using qPCR replicates to define the dynamic/quantitative range and calculate DNA copy number on the basis of positive/negative ratios (single molecule quantification; SIMQUANT).Literature cited in this table:
Berdal KG., Bøydler C, Tengs T, Holst-Jensen A. A statistical approach for evaluation of PCR results to improve the practical limit of quantification (LOQ) of GMO analyses (SIMQUANT). European Food Research and Technology 2008; 227, 1149–1157.
**Table E. Summary of the two methods used in Denmark and Norway.** Literature cited in this table:
Spens J, Evans AR, Halfmaerten D, Knudsen SW, Sengupta ME, Mak SST, et al. Comparison of capture and storage methods for aqueous macrobial eDNA using an optimized extraction protocol: advantage of enclosed filter. Methods in Ecology and Evolution. 2016;Available from: http://dx.doi.org/10.1111/2041-210X.12683.
Sigsgaard EE, Nielsen IB, Bach SS, Lorenzen ED, Robinson DP, Knudsen SW, et al. Population characteristics of a large whale shark aggregation inferred from seawater environmental DNA. Nature Ecology & Evolution. 2016 nov;1(4). Available from: http://dx.doi.org/10.1038/s41559-016-0004.
Berdal KG, Bøydler C, Tengs T, Holst-Jensen A. A statistical approach for evaluation of PCR results to improve the practical limit of quantification (LOQ) of GMO analyses (SIMQUANT). European Food Research and Technology. 2008;.
Strand DA, Jussila J, Johnsen SI, Viljamaa-Dirks S, Edsman L, Wiik-Nielsen J, et al. Detection of crayfish plague spores in large freshwater systems. Journal of Applied Ecology. 2014 4;51(2):544–553. Available from: https://dx.doi.org/10.1111/1365-2664.12218.
**Text A: Detailed description of the methodology used in this study:** Optimization of primers and probes.
**Text B: Detailed description of the methodology used in this study:** DNA extraction from tissue.
**Text C: Detailed description of the methodology used in this study:** Sequencing the mtDNA-CO1 barcode region from crayfish tissue samples, using broad range invertebrate primers.
**Text D: Detailed description of the methodology used in this study:** Specificity of each assay.
**Text E: Detailed description of the methodology used in this study:** Production of the CO1 region for standard curves.
(PDF)

S1 Appendix R-script. The supplied r-code can be executed in in R-studio: Version 0.98.994–2009–2013 RStudio, Inc.
(R) Click here for additional data file.
