# Supplementary material for: Monitoring of noble, signal and narrow-clawed crayfish using environmental DNA from freshwater samples
Source: PLoS One. 2017 Jun 27;12(6):e0179261. doi: 10.1371/journal.pone.0179261 (PMC5487031; doi:10.1371/journal.pone.0179261)
Supplement: S1 File — Contains all Supporting Figures (A-B), Tables (A-F) and Text (A-E): Figure A. Neighbour joining tree showing relationship between sequences of mtDNA-CO1 from A. leptodactylus, A. astacus and P. leniusculus. For each branch, species and accession numbers/museum catalog numbers are shown. Three clades have been collapsed: the hexagon is accession numbers JQ421496-JQ421509 within clade I, the square represents clade II and the triangle represents accession numbers KC311416, KC789374-KC789393 within clade I. The star and circle represent P. leniusculus subspecies klamathensis and trowbridgii, respectively. P. leniusculus was used as outgroup and from 1000 bootstrap pseudo replicates, relevant bootstrap values above 60% are shown. All ZMUC-CRU specimens were collected for the present study, and are listed in Table A in S1 File with Genbank accession numbers. Figure B. An alignment of the mtDNA-CO1 65 base pair fragment, used in this assay, for each of the Astacoidea and Parastacoidea present in Europe. Species and NCBI acc. number aligned using MAFFT algorithm in Geneious (Astacus astacus JN254670, JN254671; Astacus leptodactylus Clade I JQ421471, JQ421471; A. leptodactylus Clade II JQ421478, JQ421479; A. leptodactylus Clade III JQ421489, JQ421490; Austropotamobius torrentium AY667128, AM180946; A. italicus HM622614, AY121127; A. pallipes AY667114, AY667115; Cherax destructor KM039112, KJ950555; Orconectes immunis JF438005, JF438006; O. limosus JF437992, JF437993; O. virilis FJ608577, EU442743; O. rusticus AY701248, AY701249; Pacifastacus leniusculus JF437995, JF437995; Procambarus clarkii JN000900, JN000901; Procambarus sp. HM358011, KF033123). The species-specific primers and probes used in this study are mapped to the sequences where they have the best match. Forward primers (F0336) Reverse primers (R0397) are green and probes (P0357) are red. Agreements to consensus are marked with dots and disagreements to consensus are highlighted. The specific number of mismatches [file pone.0179261.s001.pdf]

Supporting Information for:

Monitoring of noble, signal and narrow-clawed crayfish using environmental DNA from freshwater samples

Sune Agersnap[1,*,†], William Brenner Larsen[1,†], Steen Wilhelm Knudsen[1], David Strand[2], Philip Francis Thomsen[3], Martin Hesselsøe[4], Peter Bondgaard Mortensen[5], Trude Vrålstad[2], Peter Rask Møller[1]

1) Section for Evolutionary Genomics, Natural History Museum of Denmark, University of Copenhagen, Universitetsparken 15, DK-2100 Copenhagen Ø, Denmark

2) Norwegian Veterinary Institute, Pb 750 Sentrum, N-0106 Oslo, Norway

3) Centre for GeoGenetics, Natural History Museum of Denmark, University of Copenhagen, Øster Voldgade 5-7, DK-1350 Copenhagen, Denmark.

4) Amphi Consult ApS, Niels Jernes Vej 10, DK-9220 Aalborg Øst, Denmark.

5) Eurofins Miljø A/S, Ladelundsvej 85, DK-6600 Vejen, Denmark

*) Corresponding author: [suneagersnap@gmail.com]

†) These authors contributed equally to this work

Figure A: Neighbour joining tree showing relationship between sequences of *mtDNA-CO1* from *A. leptodactylus*, *A. astacus* and *P. leniusculus*.

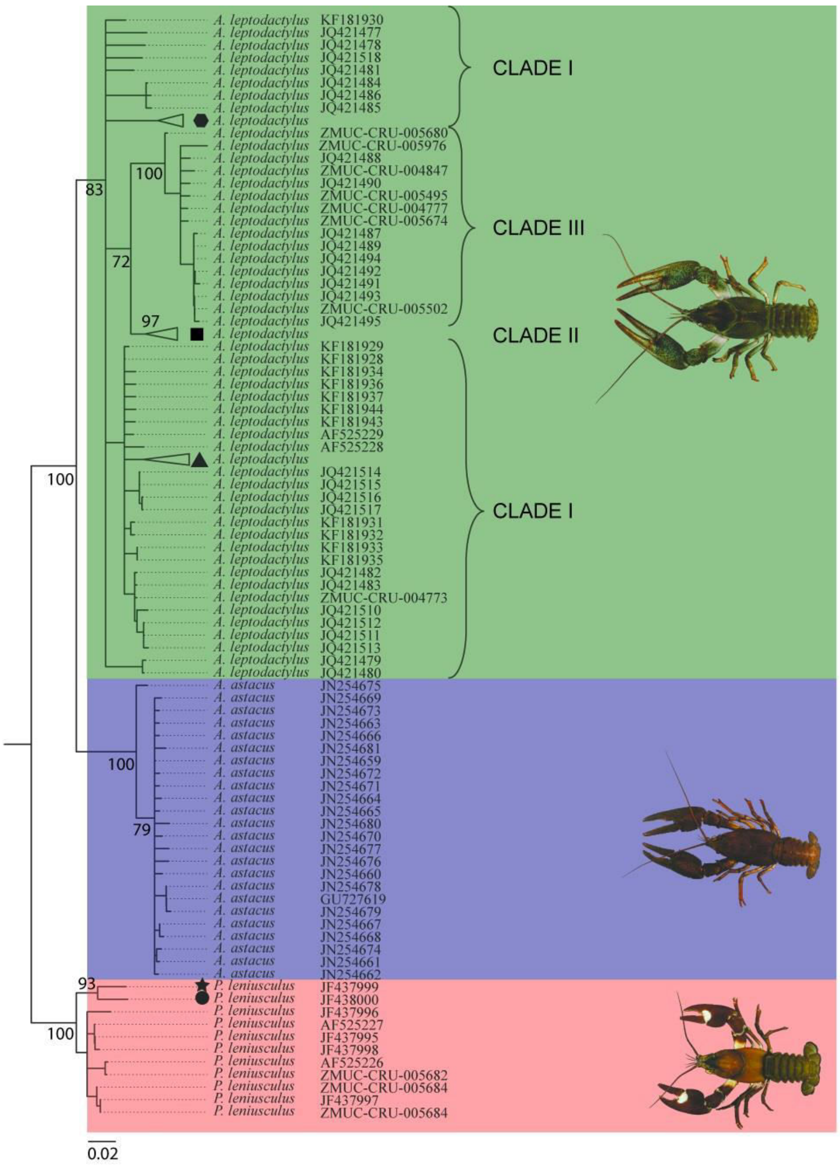

Figure B: Species-specific primer-probe assays for *Astacus astacus*, *Pacifastacus leniusculus* and *Astacus leptodactylus* with number of mismatch in the alignment with various other species.

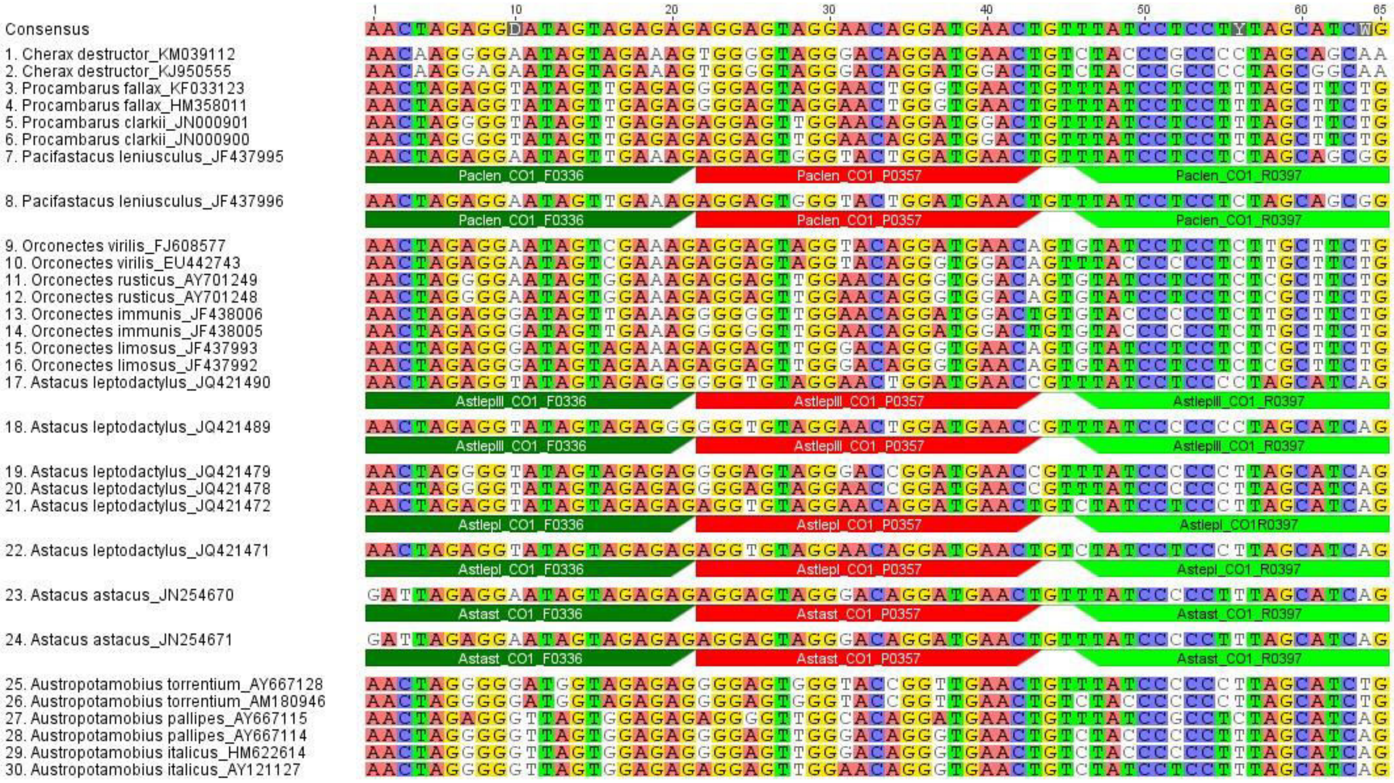

Figure C: Standard curve for dilution series and filtered water samples comparing the concentration of eDNA target (copies/qPCR reaction) with cycle threshold (Ct). The approach used by University of Copenhagen.

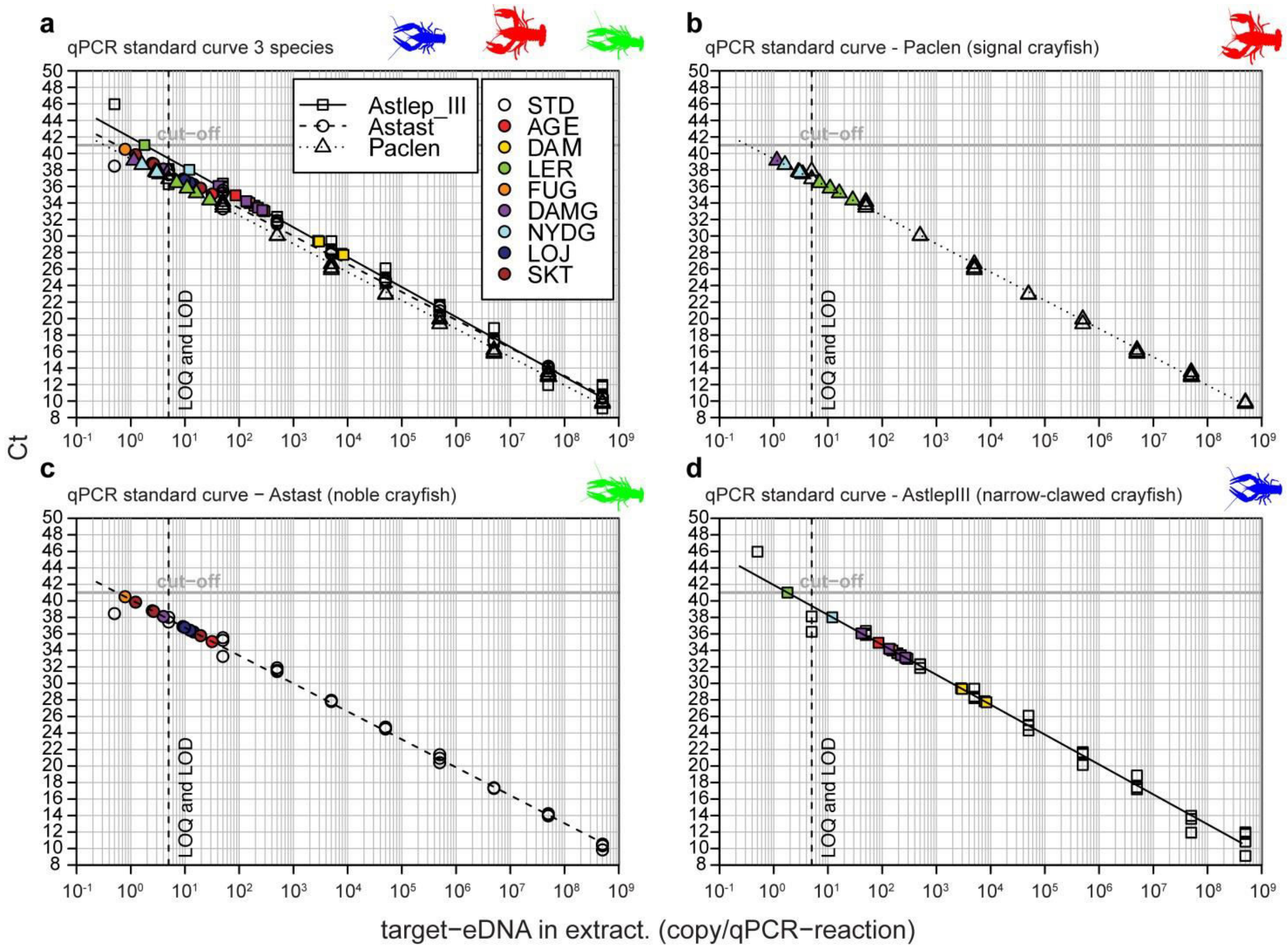

Figure D: Standard curves for dilution series and filtered water samples comparing the concentration of eDNA target (copies/qPCR reaction) with cycle threshold (Ct). The approach used by Norwegian Veterinary Institute.

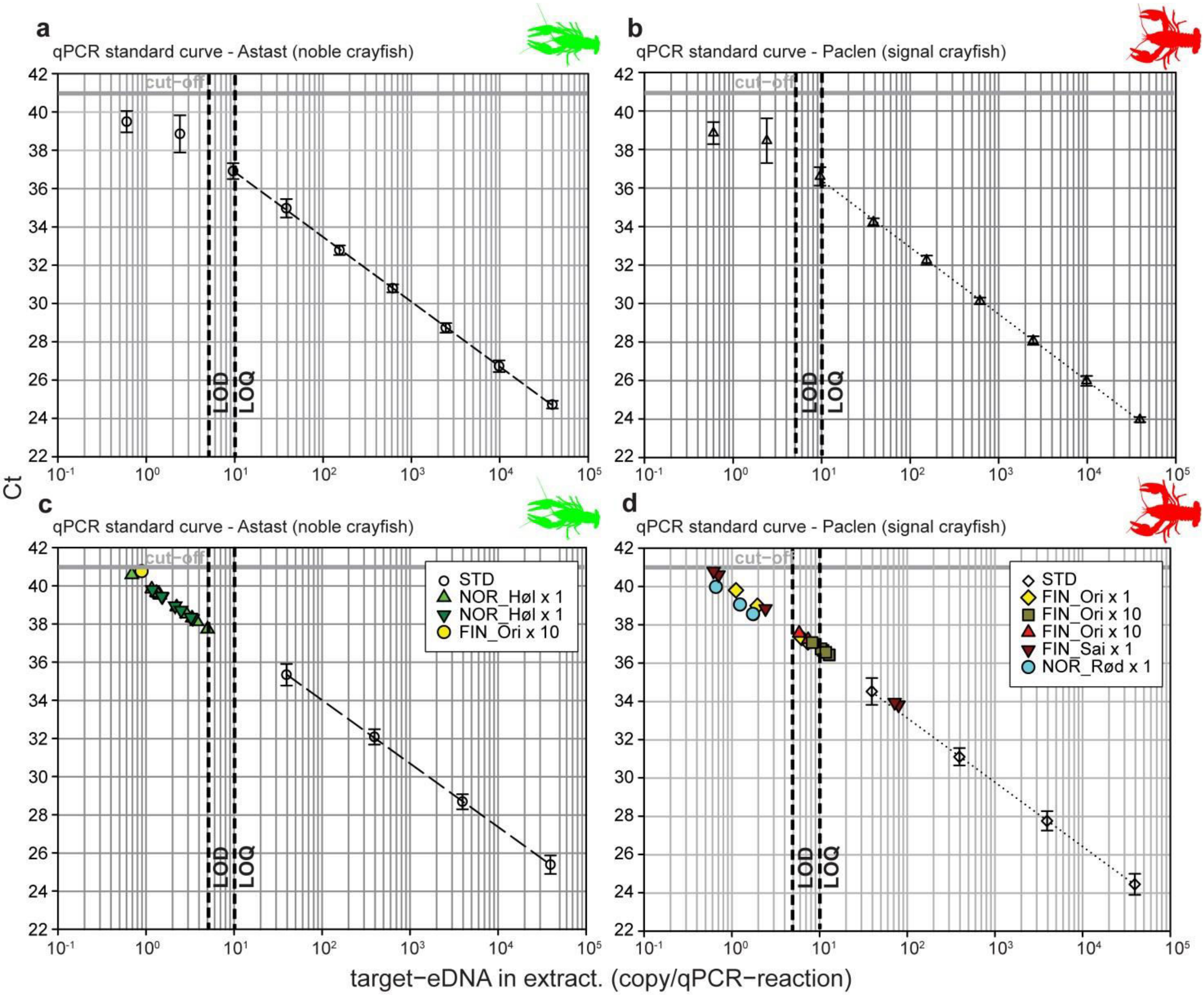

Table A: Danish collected vouchered museum specimens.

Species	Sample site (longitude-latitude)	Code	Type	ZMUC, ID.	NCBI accession number
Astacus leptodactylus	12.438907-55.691911	VOL	Channel	ZMUC-CRU-004773	MF288079
Astacus leptodactylus	12.457318-55.689385	SCH	Pond	ZMUC-CRU-004847	MF288080
Astacus leptodactylus	12.357194-55.760575	SKA	Pond	ZMUC-CRU-004777	MF288081
Astacus leptodactylus	12.653911-55.640496	KAS	Brackish	ZMUC-CRU-005976	MF288082
Astacus leptodactylus	12.480795-55.672899	DAM	Lake	ZMUC-CRU-005674	MF288083
Astacus leptodactylus	12.351023-55.640567	VAL	Lake	ZMUC-CRU-005495	MF288084
Astacus leptodactylus	12.657066-55.638314	DBP	Pond	ZMUC-CRU-005680	MF288085
Astacus leptodactylus	12.420731-55.796842	FUR	Lake	ZMUC-CRU-005502	MF288086
Astacus astacus	12.378904-56.042448	ESR	Stream	ZMUC-CRU-005649	
Astacus astacus	12.558574-55.681184	SKJ	Lake	ZMUC-CRU-004845	
Astacus astacus	12.305374-55.822068	LYN	Lake	ZMUC-CRU-004810	
Astacus astacus	12.186253-55.636285	HED	Lake	ZMUC-CRU-005699	
Astacus astacus	12.345997-55.7477	MAR	Lake	ZMUC-CRU-004844	
Astacus astacus	12.305374-55.822068	LYN	Lake	ZMUC-CRU-004809	
Astacus astacus	12.558574-55.681184	SKJ	Lake	ZMUC-CRU-004846	
Astacus astacus	12.378904-	ESR	Stream	ZMUC-CRU-	

	56.042448			005695	
Pacifastacus leniusculus	12.442784-55.769896	NYD	Lake	ZMUC-CRU-005691	
Pacifastacus leniusculus	12.442784-55.769896	NYD	Lake	ZMUC-CRU-005684	MF288087
Pacifastacus leniusculus	12.442784-55.769896	NYD	Lake	ZMUC-CRU-005683	
Pacifastacus leniusculus	12.152816-55.442244	HAS	Lake	ZMUC-CRU-005682	MF288088
Pacifastacus leniusculus	12.442784-55.769896	NYD	Lake	ZMUC-CRU-005684	MF288089
Pacifastacus leniusculus	12.442784-55.769896	NYD	Lake	ZMUC-CRU-005685	
Pacifastacus leniusculus	12.442784-55.769896	NYD	Lake	ZMUC-CRU-005691	
Pacifastacus leniusculus	12.293014-55.564369	MOS	Brackish	ZMUC-CRU-006036	

Table B: Sample sites and Genbank references for crayfish used in this manuscript.

Species	Country/US states	Locality	Accession number Genbank	References
Astacus leptodactylus	Turkey,	Lake Egirdir	KC311416	Keskin and Atar 2013
Astacus leptodactylus	Croatia	(Dobra)	KF181928, KF181929	Maguire et al. 2014
Astacus leptodactylus	Croatia	(Mrežnica)	KF181932, KF181937	Maguire et al. 2014
Astacus leptodactylus	Croatia	(Odra)	KF181933	Maguire et al. 2014
Astacus leptodactylus	Croatia	(Una)	KF181930, KF181931	Maguire et al. 2014
Astacus leptodactylus	Bulgaria	Unknown	KF181943, KF181944	Maguire et al. 2014
Astacus leptodactylus	Poland	(Gaj lake)	AF525228, AF525229	Maguire et al. 2014
Astacus leptodactylus	Turkey		JQ623972	Maguire et al. 2014
Astacus leptodactylus	Russia	(Siberia, Tyumen)	KF181941, KF181942	Maguire et al. 2014
Astacus leptodactylus	Armenia	(Unknown)	KF181938-KF181940, KF181945-KF181954	Maguire et al. 2014
Astacus leptodactylus	Turkey	Altinyazi Dam Lake	JQ421465-JQ421468	Akhan et al. 2014
Astacus leptodactylus	Turkey	Kadikoy Dam Lake	JQ421465, JQ421466 JQ421468-JQ421474	Akhan et al. 2014
Astacus leptodactylus	Turkey	Karpuzlu Dam Lake	JQ421465, JQ421468JQ421469, JQ421471, JQ421474	Akhan et al. 2014
Astacus leptodactylus	Turkey	Lake Iznik	JQ421506-JQ421509	Akhan et al. 2014
Astacus leptodactylus	Turkey	Lake Kucukcekmece	JQ421465JQ421466JQ421468JQ421469	Akhan et al. 2014
Astacus leptodactylus	Turkey	Lake Apolyont	JQ421482JQ421510	Akhan et al. 2014
Astacus leptodactylus	Turkey	Lake Manyas	JQ421496JQ421500	Akhan et al. 2014
Astacus leptodactylus	Turkey	Lake Hamam	JQ421514JQ421517	Akhan et al. 2014
Astacus leptodactylus	Turkey	Pabucdere,	JQ421518	Akhan et al. 2014
Astacus leptodactylus	Turkey	Terkos Dam Lake	JQ421511-JQ421513	Akhan et al. 2014
Astacus leptodactylus	Turkey	Lake Golmarmara	JQ421482JQ421483	Akhan et al. 2014

Astacus leptodactylus	Turkey	Lake Egirdir	JQ421477-JQ421481JQ421501-JQ421505	Akhan et al. 2014
Astacus leptodactylus	Turkey	Lake Karatas		Akhan et al. 2014
Astacus leptodactylus	Turkey	Lake Balik	JQ421487-JQ421492	Akhan et al. 2014
Astacus leptodactylus	Turkey	Lake Sera	JQ421488JQ421489JQ421493-JQ421495	Akhan et al. 2014
Astacus leptodactylus	Turkey	Keban Dam Lake	JQ421482-JQ421486	Akhan et al. 2014
Astacus leptodactylus	Turkey	Lake Cildir	JQ421475JQ421476	Akhan et al. 2014
Astacus leptodactylus	Turkey	Velika	JQ421519JQ421520	Akhan et al. 2014
Astacus leptodactylus	Turkey	Lake Egirdir	KC789374-KC789393	Keskin and Atar 2013
Astacus astacus	Croatia		GU727619	Jadan et al. 2010
Astacus astacus	Austria, Bulgaria, Croatia, Germany, Hungary, Romania, Czech Republic, Belgium and Poland		JN254659-JN254681	Schrimpf et al. 2011
Pacifastacus leniusculus	Czech republic	Jedlova, Litomysl	JF437995	Filipova et al. 2011
Pacifastacus leniusculus	Hungary	Gyongyos, Koszeg	JF437996	Filipova et al. 2011
Pacifastacus leniusculus	Great Britain	Teil Burn, Five	JF437997	Filipova et al. 2011
Pacifastacus leniusculus	Oregon	Upper Williamson River	JF437998	Filipova et al. 2011
Pacifastacus leniusculus	California	Lower Klamath River	JF437999	Filipova et al. 2011
Pacifastacus leniusculus	Oregon	Suislaw river	JF438000	Filipova et al. 2011
Pacifastacus leniusculus	Poland		AF525226-AF525227	Soroka et al. 2002

Table C: Primer- and probe sequence together with number of mismatch in the alignment (Algn) with various other species of Astacoidea and Parastacoidea. The average of the nucleotide differences from two *mtDNA-CO1* sequences for each of these 15 species from NCBI Genbank (*Astacus astacus* JN254670, JN254671; *Astacus leptodactylus* Clade I JQ421471, JQ421471; *A. leptodactylus* Clade II JQ421478, JQ421479; *A. leptodactylus* Clade III JQ421489, JQ421490; *Austropotamobius torrentium* AY667128, AM180946; *A. italicus* HM622614, AY121127; *A. pallipes* AY667114, AY667115; *Cherax destructor* KM039112, KJ950555; *Orconectes immunis* JF438005, JF438006; *O. limosus* JF437992, JF437993; *O. virilis* FJ608577, EU442743; *O. rusticus* AY701248, AY701249; *Pacifastacus leniusculus* JF437995, JF437995; *Procambarus clarkii* JN000900, JN000901; *Procambarus* sp. HM358011, KF033123). The forward-primer (F), the reverse-primer (R), and the probe (P). For species specific detection of *Astacus astacus*, *Pacifastacus leniusculus*, *Astacus leptodactylus*, clade I and *Astacus leptodactylus*, clade III, the following primer sets were developed: Astast_COI_F0336 (5'-GATTAGAGGAATAGTAGAGAG-3'), Astast_COI_R0397 (5'-CTGATGCTAAAGGGGGATAA-3'), Paclen_COI_F0336 (5'-AACTAGAGGAATAGTTGAAAG-3'), Astlen_COI_R0397 (5'-CCGCTGCTAGAGGAGGATAA-3'), AstlepI_COI_F0336 (5'-AACTAGGGGTATAGTAGAGAG-3'), AstlepI_COI_R0397 (5'-CTGATGCTAAAGGGGGATAA-3'), AstlepIII_COI_F0336 (5'-AACTAGAGGTATAGTAGAGGG-3') and AstlepIII_COI_R0397 (5'-CTGATGCTAGGGGAGGATAA-3'). For each of these four primer sets the following species specific BHQ-1-probes with FAM-modifications were prepared: Astast_COI_P0357 (5'-FAM-AGGAGTAGGGACAGGATGAACT-BHQ-1-3'), Paclen_COI_P0357 (5'-FAM-AGGAGTGGGTACTGGATGAACT-BHQ-1-3'), AstlepI_COI_P0357 (5'-FAM-AGGAGTAGGGACCGGATGAACT-BHQ-1-3') and AstlepIII_COI_P0357 (5'-FAM-GGGTGTAGGAACTGGATGAACC-BHQ-1-3').

**Primer-probe systems**		***Astast***				***Paclen***				***AstlepI***				***AstlepIII***		
**Oligo-name**		**Astast_COI_F0336**	**Astast_COI_R0397**	**Astast_COI_P0357**		**Paclen_COI_F0336**	**Paclen_COI_R0397**	**Paclen_COI_P0357**		**AstlepI_COI_F0336**	**AstlepI_COI_R0397**	**AstlepI_COI_P0357**		**AstlepIII_COI_F0336**	**AstlepIII_COI_R0397**	**AstlepIII_COI_P0357**
**Species**	**Algn**	**F**	**R**	**P**	**Algn**	**F**	**R**	**P**	**Algn**	**F**	**R**	**P**	**Algn**	**F**	**R**	**P**
Astacus astacus	0	0	0	0	11	4	4	3	8	3	3	2	12	4	2	5
Pacifastacus leniusculus	11	4	4	3	0	0	0	0	12	3	5	4	11	4	4	5
Astacus leptodactylus, cl. I	8	3	3	2	12	3	5	4	0	0	0	0	6	1	3	3
Astacus leptodactylus, cl. II	8	4	1	4	14	4	5	5	7	1	2	4	6	2	1	2
Astacus leptodactylus, cl. III	11	4	2	5	11	4	4	5	6	1	3	3	1	0	0	0
Austropotamobius torrentium	13	5	2	5	14	5	5	3	12	3	3	6	13	4	2	6
Austropotamobius italicus	11	6	2	3	14	5	6	5	10	4	3	5	15	5	4	6
Austropotamobius pallipes	10	6	2	3	13	5	6	5	11	4	3	5	14	5	4	6
Cherax destructor	15	5	7	2	15	3	6	5	13	4	5	3	13	5	5	5
Orconectes immunis	16	5	6	5	14	1	7	6	15	3	8	4	15	4	6	5
Orconectes limosus	13	4	6	3	12	2	5	5	13	2	6	5	15	3	6	7
Orconectes virilise	12	4	5	4	10	1	6	5	14	3	8	5	15	4	5	7
Orconectes rusticus	16	5	6	5	13	2	5	6	15	4	6	5	17	5	6	7
Procambarus clarkii	11	5	3	3	11	3	4	4	9	2	4	3	13	3	5	6
Procambarus sp.	11	5	3	3	10	3	4	4	9	2	4	3	9	3	5	6

Table D: Standard dilutions from genomic crayfish DNA. Standard curves were established from several calibrant points using qPCR replicates to define the dynamic/quantitative range and to calculate DNA copy number on the basis of positive/negative ratios (single molecule quantification; SIMQUANT).

Standard dilutions[a]	Mean Ct-values (n = 10)[b]	% detection[c]	DNA (ng/µl) in calibrants[d]	ng DNA (2 µl) in PCR[e]	Estimated DNA copies/PFU in PCR[f]
Noble crayfish					
4^3	24.71 (±0.20)	100	7.81×10^{-1}	1.56	39321.6
4^4	26.72 (±0.30)	100	1.95×10^{-1}	3.91×10^{-1}	9830.4
4^5	28.72 (±0.25)	100	4.88×10^{-2}	9.77×10^{-2}	2457.6
4^6	30.79 (±0.21)	100	1.22×10^{-2}	2.44×10^{-2}	614.4
4^7	32.78 (±0.25)	100	3.05×10^{-3}	6.10×10^{-3}	153.6
4^8	34.97 (±0.48)	100	7.63×10^{-4}	1.53×10^{-3}	38.4
4^9	36.91 (±0.41)	100	1.91×10^{-4}	3.81×10^{-4}	9.6
4^10	38.85 (±0.97)	90.9	4.77×10^{-5}	9.54×10^{-5}	2.4
4^11	39.43 (±0.56)	37.5	1.19×10^{-5}	2.38×10^{-5}	0.6
Signal crayfish					
4^3	23.96 (±0.13)	100	7.81×10^{-1}	1.56	39321.6
4^4	25.98 (±0.26)	100	1.95×10^{-1}	3.91×10^{-1}	9830.4
4^5	28.07 (±0.22)	100	4.88×10^{-2}	9.77×10^{-2}	2457.6
4^6	30.13 (±0.17)	100	1.22×10^{-2}	2.44×10^{-2}	614.4
4^7	32.25 (±0.24)	100	3.05×10^{-3}	6.10×10^{-3}	153.6
4^8	34.23 (±0.20)	100	7.63×10^{-4}	1.53×10^{-3}	38.4
4^9	36.60 (±0.48)	100	1.91×10^{-4}	3.81×10^{-4}	9.6
4^10	38.46 (±1.16)	90.9	4.77×10^{-5}	9.54×10^{-5}	2.4
4^11	38.85 (±0.57)	41.7	1.19×10^{-5}	2.38×10^{-5}	0.6

[a] In total of 11 standard dilutions were made from a four-fold dilution series where the stock solutions contained 50 ng/ µL genomic DNA from respectively noble and signal crayfish. A standard curve was established from several calibrant points (standard dilutions from 4^3 - 4^9), cf. Fig 3a-b.

[b] Mean Ct-values are based on the qPCR replicates of each standard. The standards from the 3rd to the 9th dilution were run in 10 replicates, while the 10th and 11th dilution in 22 and 24, respectively, to get a larger sample for positive/negative ratio.

[c] The percentage of RT-PCR replicates yielding positive results (detection) for each standard.

[d] Theoretical content of DNA in ng/ µL for each standard calculated from the concentration assigned to the DNA stock (50 ng/ml).

[e] Quantity of template DNA in each qPCR replicate in (2 µL template multiplied by assigned concentration per µL).

[f] Number of detected DNA copies in each PCR replicate (2 µL template DNA) estimated on the basis of application of single molecule quantification (SIMQUANT; Berdal et al.. 2008).

Table E: Summary of the two methods used in Denmark and Norway.

		**Denmark**	**Norway and Finland**
**Water sampling**	Filter type	Sterivex^{TM}-GP filter unit	EMD AP25 Millipore glass fiber filters
	Pore size	0.22 µm	2 µm
	Volume filtered per filter	0.5 - 1.5 L	0.5 - 14 L
	Biological filter replicates	1	3
	Sampling method	Hand held syringe, single use, disposables.	Mechanical pump, multiple use, decontaminated by bleaching.
**Extraction from filters**	Extraction kit	Qiagen DNeasy Blood & Tissue kit with minor modifications as in Spens et al. 2016.	Large volume DNA extraction using CTAB as in Strand et al. 2014.
**qPCR quantification**	Standard dillution	Based on purified target-DNA as described by Sigsgaard et al. 2016.	Based on genomic DNA, using SIMQUANT as described by Berdal et al. 2008.

Text A: Optimization of primers and probes.

Primer and probe concentrations were optimized for the *Astast* and *Paclen* assays by testing qPCR reaction setups with forward and reverse primer in the following reaction concentrations: 200, 400, 600, 800, 1000 and 1200 nM. Following Bustin et al. [36] the concentrations resulting in the lowest cycle threshold value (Ct) for each assay were considered the optimal setting – i.e. optimal concentration of primers. These optimized primer concentrations were applied in the subsequent tests of the optimal probe concentration for each of the assays. Probe reaction concentrations of 100, 200, 300 400 and 500 nM were tested on DNA tissue extractions from the target species. Results were extrapolated to both the *AstlepI*- and the *AstlepIII* assay and adopted in subsequent qPCR-tests performed on extractions of eDNA from filtered water (*in situ*).

Text B: DNA extraction from tissue.

Approximately 100mg of tissue (one crayfish walking leg) was homogenised with 500 ATL buffer using Percellys® MK28 tubes and Precellys® 24 homogenisator. The samples were frozen at -80 °C and then thawed at 56 °C. The samples were incubated at 56 °C for 30 min. with 10µl RNAse (10mg/ml), followed by 30 min. incubation with 10µl Protinase K (20mg/ml) at 56 °C. After centrifugation (5min at 10000g), 200µL of the supernatant was transferred to new tubes and DNA was extracted on the automated QIAcube, using the Tissue DNA mini protocol. The DNA was eluded in 100µl AE buffer.

Text C: Sequencing the mtDNA-CO1 barcode region from crayfish tissue samples, using broad range invertebrate primers.

Reactions were prepared in linear PCR setups with reaction volumes of 25 µL, containing 1.25 µL forward primer (10 µM), 1.25 µL reverse primer (10 µM), 0.1 µL of AmpliTaq Polymerase (5 U/µL) (Thermo Fisher Scientific), 2.5 µL dNTPs (2 mM), 2.5 µL 10 x Buffer (AmpliTaq), 15.4 µL ddH_2O and 2 µL of DNA-template extracted from tissue.

Thermal conditions were set with an initial 2 minutes at preheat 95 ºC, followed 30 cycles of (95 ºC for 30 s, 56 ºC for 30 s, and 72ºC for 1 minute) and a final extension at 72 ºC for 5 minutes. Resulting amplicons were checked on a 2% agarose gel stained with GelRed (Thermo Fisher Scientific).

Text D: Specificity of each assay.

Reaction volume of 25.0 µL, containing 2.0 µL genomic DNA template extracted from tissue, 10.0 µL of 2.0 U/µL TaqMan® Environmental Master Mix 2.0 (Life Technologies) 1.0 µL of 10.0 µM of each primer (forward and reverse), 1.0 µL of 2.5 µM TaqMan®-probe with 5'-Fam- and –BHQ1-3'-end modification [35], and 10.0 µL ddH_2O.

Thermal conditions were split in to two phases. Phase 1 comprised an initial preheat at 50°C for 5 minutes, followed by 10 minutes at 95°C. Phase 2 comprised 50 cycles of 95°C for 30 s and 60°C for 1 minute, with end-point collections of fluorescence at the 60°C step.

Text E: Production of the CO1 region for standard curves.

Each sample was prepared as a 25 µL reaction volume containing 2 µL extracted DNA template, 0.1 µL (5 U/µL) of AmpliTaq Polymerase (Applied Biosystems), 2.5 µL dNTPs (2 mM each), 2.5 µL x10 buffer (AmpliTaq), 1.25 µL of each of the species-specific primers (10 µM) and 15.4 µL ddH_2O.

Thermal conditions were split into three phases. Phase 1 comprised an initial denaturation at 95 °C for 1 minute. Phase 2 comprised 35 cycles of denaturation at 95°C for 30 s, annealing at 60°C for 45 s and extension at 72°C 90 s. Phase 3 is final extension at 72°C for 5 minutes.
